# Predicting COVID-19 Transmission to Inform the Management of Mass Events: Model-Based Approach

**DOI:** 10.2196/30648

**Published:** 2021-12-01

**Authors:** Claire Donnat, Freddy Bunbury, Jack Kreindler, David Liu, Filippos T Filippidis, Tonu Esko, Austen El-Osta, Matthew Harris

**Affiliations:** 1 Department of Statistics University of Chicago Chicago, IL United States; 2 Department of Plant Biology Carnegie Institution for Science Stanford, CA United States; 3 Faculty of Medicine, School of Public Health Imperial College London United Kingdom; 4 Institute of Genomics University of Tartu Tartu Estonia

**Keywords:** COVID-19, transmission dynamics, live event management, Monte Carlo simulation

## Abstract

**Background:**

Modelling COVID-19 transmission at live events and public gatherings is essential to controlling the probability of subsequent outbreaks and communicating to participants their personalized risk. Yet, despite the fast-growing body of literature on COVID-19 transmission dynamics, current risk models either neglect contextual information including vaccination rates or disease prevalence or do not attempt to quantitatively model transmission.

**Objective:**

This paper attempted to bridge this gap by providing informative risk metrics for live public events, along with a measure of their uncertainty.

**Methods:**

Building upon existing models, our approach ties together 3 main components: (1) reliable modelling of the number of infectious cases at the time of the event, (2) evaluation of the efficiency of pre-event screening, and (3) modelling of the event’s transmission dynamics and their uncertainty using Monte Carlo simulations.

**Results:**

We illustrated the application of our pipeline for a concert at the Royal Albert Hall and highlighted the risk’s dependency on factors such as prevalence, mask wearing, and event duration. We demonstrate how this event held on 3 different dates (August 20, 2020; January 20, 2021; and March 20, 2021) would likely lead to transmission events that are similar to community transmission rates (0.06 vs 0.07, 2.38 vs 2.39, and 0.67 vs 0.60, respectively). However, differences between event and background transmissions substantially widened in the upper tails of the distribution of the number of infections (as denoted by their respective 99th quantiles: 1 vs 1, 19 vs 8, and 6 vs 3, respectively, for our 3 dates), further demonstrating that sole reliance on vaccination and antigen testing to gain entry would likely significantly underestimate the tail risk of the event.

**Conclusions:**

Despite the unknowns surrounding COVID-19 transmission, our estimation pipeline opens the discussion on contextualized risk assessment by combining the best tools at hand to assess the order of magnitude of the risk. Our model can be applied to any future event and is presented in a user-friendly RShiny interface. Finally, we discussed our model’s limitations as well as avenues for model evaluation and improvement.

## Introduction

### Background

#### Evaluating the Safety of Live Events

More than a year after a global and unprecedented cancellation of live events in March 2020, the future of live events and the entertainment industry remains uncertain despite increasing vaccination rates and low community prevalence levels (at the time of writing). The main concern raised by these gatherings lies in their susceptibility to “super-spreading”—a scenario whereby a few contagious participants inadvertently infect a disproportionately large number of others [1-6] and that has been highlighted as a significant driver of the pandemic [7-10]. Despite the re-opening of live events in the United Kingdom on July 19, 2021, the threat of existing and emergent COVID-19 variants coupled with dwindling immunity from vaccination over time suggests that policy makers and event organizers will likely continue to struggle with the following 2 questions: (1) Is the COVID-19 transmission risk posed by these events tolerable? and (2) What additional safety measures can be feasibly deployed to reduce this risk?

The answer to these questions is inherently tied to the estimation of 2 quantities: the number of infections occurring at the event and the postevent secondary attack rate, or number of subsequent infections in the participants’ social circles. Evaluating the safety (or lack thereof) of large public gatherings can then be reframed as quantifying the significance and magnitude of their effect on the distribution of the number of primary and secondary COVID-19 cases. Yet, despite the growing body of literature on COVID-19 risk evaluation and recent efforts to evaluate the safety of live events, this effect remains ill-characterized. Nevertheless, over the past several months, several calculators were developed to estimate this risk [11-14]. These methods can typically be placed in 1 of 3 categories: ranking heuristics, context-based heuristics, and transmission risk calculators.

#### Ranking Heuristics

These estimators typically rank events on a scale ranging from “low” risk to “high” risk based on the feedback of medical experts [13,15-17]. However, these heuristics do not take into account contextual information, including the prevalence. For example, the risk associated with an event would be classified as high regardless of whether it was held in August 2020 (background prevalence of 1 in 3000 individuals in the United Kingdom) or January 2021 (prevalence of 1 in 60 individuals [18]).

#### Context-Based Heuristics

These calculators estimate the probability of encountering 1 COVID-19 case based on the number of people attending an event [11,12]. While more context-aware than risk assessment charts, such estimators do not attempt to model transmission dynamics—which is undeniably one of the main unknowns in the spread of viral epidemics—and consequently rarely stratify risk by type of activity. To exemplify, a classical music recital of 1.5 hours for the BBC Proms would potentially be considered equally risky to a 3-hour concert in which participants could be expected to sing along.

#### Transmission Risk Calculators

Stemming from physics or fluid dynamics, these calculators focus on modelling the aerosolization and spread of microdroplets—typically in a closed or indoor environment [19-22]. These fine-grained models thus must be combined with extensive and often prohibitive simulations of crowd movements in order to model transmission dynamics during any given event.

#### Limitations of Existing Estimators

Regardless of their category, most of these models rely on a large number of input parameters, including (but not restricted to) the prevalence of the disease. While certain calculators attempt to bridge the gap between expert heuristics and physical models [11,23], they are not capable of predicting the risk of a future event. Moreover, all of these estimators provide point estimates—in other words, their output is a single number to quantify the risk. Given the uncertainty associated with all the inputs and the parametrization of the problem as well as the high stochasticity of viral transmission, the provision of a single consolidated outcome or number can potentially be misleading. This is because a singular focus on the expected outcome precludes consideration of the distribution of all possible outcomes, including worst-case scenarios. In the context of COVID-19, where the majority of new cases has been shown to be caused by a minority of index cases [24-26], the modelling of tail events and potential super-spreader phenomena takes on significant importance for risk assessment [26,27].

### Mitigating Transmission Risk

Meanwhile, with the increasing vaccination rates in several countries around the world, a few initiatives have begun to evaluate the outbreak risk associated with live events empirically [28-31]. This is because vaccinated individuals may still be infected with SARS-CoV-2 [32,33], and even antigen-test based screening of ticket holders offers no guarantee due to false negatives [34,35]. The estimation of what constitutes an admissible level of risk thus poses a difficult conundrum to the live event industry. To begin answering these questions, the CAPACITY study [36]—a partnership between Certific (a private, remote testing, health status, and identify certification service) and Imperial College London—aims to predict and measure the outcomes of full capacity live events while ensuring rigorous implementation and alignment to current public health and recommended safety measures. Central to this study is the provision of a streamlined and efficient pre-event screening protocol of all ticket holders using professionally witnessed rapid at-home antigen tests followed by postevent monitoring based on antigen tests, surveys, and safety recommendations (see Multimedia Appendix 1). In this setting, providing risk estimates not only becomes essential in communicating to the ticket holders their own level of risk so that they may make an informed decision of whether to attend the event but also necessary to inform event managers and policy makers on the likelihood of an outbreak task that serves here as the motivating application behind this paper.

### A Working Example: Concert at the Royal Albert Hall

In order to understand and illustrate the potential challenges that arise in the risk estimation for the CAPACITY study, we considered as an example a concert at the Royal Albert Hall (RAH) and demonstrate how to estimate the associated risk assuming a near capacity attendance of 5000 in the main concert hall, which has a volume of 86,650 m^3^ [37], with a dwell time of 3 hours. Attendees will be assumed to be a cross-section representative of the general British public and will be required to have a negative COVID-19 antigen test result within 2 days prior to the event, as well as satisfying other self-declared symptoms and exposure-risk questions. Vaccination status would be requested, but not required, for attendance, and full compliance with mask wearing was assumed in our default example.

### Goals and Contributions

**Figure 1:**
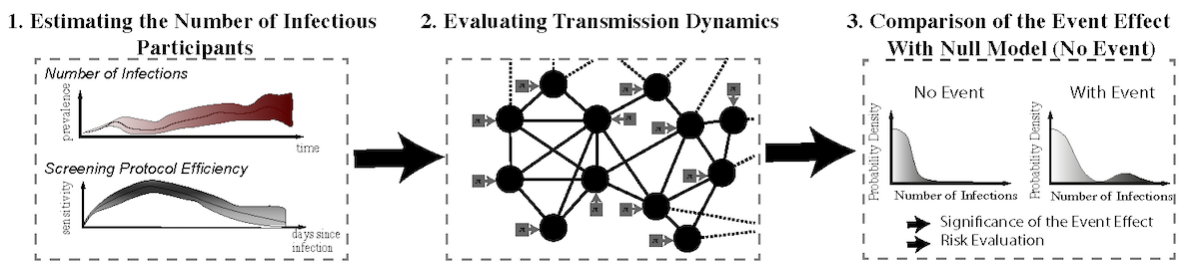
Summary of our modelling pipeline.

The objectives of our modelling approach were threefold: (1) enable the quantitative comparison of different activities and event characteristics, (2) estimate the efficacy of various safety protocols, and (3) provide a predictive risk assessment (ie, the risk associated with a scheduled future event). To this end, we delineated our approach into 3 sequential steps (see Figure 1): (1) estimating the number of contagious participants, (2) evaluating the transmission dynamics, and (3) comparing the risk of holding the event with the null model (ie, if the event had not taken place). We illustrated the application of our risk modelling pipeline in the RAH example to highlight the risk’s dependency on factors such as prevalence, mask wearing, number of attendees, and event duration. In particular, we demonstrated how this particular event held on 3 different dates corresponding to 3 distinct COVID-19 prevalence regimes in the United Kingdom (stable low prevalence: August 20, 2020; high prevalence peak: January 20, 2021; medium declining prevalence: March 20, 2021) would likely lead to transmission events that were on par with community transmission rates (0.06 vs 0.07, 2.38 vs 2.39, and 0.67 vs 0.60, respectively; see Table 1). However, the 99th percentile of the prediction interval for the infections at the event would likely be substantially higher than the background rate (1 vs 1, 19 vs 8, and 6 vs 3, respectively), further demonstrating that sole reliance on vaccination and antigen testing to gain entry would significantly underestimate the tail risk of the event. However, we emphasize that the goal of this paper is not to present a novel “state-of-the-art” risk estimation procedure. This is because COVID-19 transmission mechanisms remain poorly characterized, and we acknowledge that our approach requires certain simplifications and assumptions that we discuss at length in the last section of this paper. Rather, faced with the need to provide a risk evaluation tool despite many unknowns, our estimation pipeline combined the best tools at hand to assess the order of magnitude of the risk—thereby opening the avenue for further work on contextualized COVID-19 risk assessment. Consequently, in providing a pipeline for risk estimation, our objective was twofold: (1) developing a publicly available platform to increase risk awareness and promote informed consent for event organizers and participants, while simultaneously (2) encouraging the data collection that is currently so desperately needed for risk assessment. Our model can be applied to any event occurring in the near future and is presented in a user-friendly RShiny interface [38].

**Table 1:** Quantiles of the number of transmission events for the Royal Albert Hall concert, by event date, assuming that all participants were wearing masks, so that the exhalation of particles is reduced by 70% and inhalation by 50%.

Statistics	August 20, 2020		January 20, 2021		March 20, 2021		
	Event	Null	Event	Null		Event	Null
Median	0	0	1	2		0	0
Mean	0.06	0.07	2.38	2.49		0.67	0.63
1st percentile	0	0	0	0		0	0
2.5th percentile	0	0	0	0		0	0
97.5th percentile	1	1	10	7		3	3
99th percentile	1	1	19	8		6	3

## Methods

### Modelling the Risk of a Large Public Event

#### Step 1: Estimating the Number of Infectious Participants

**Figure 2:**
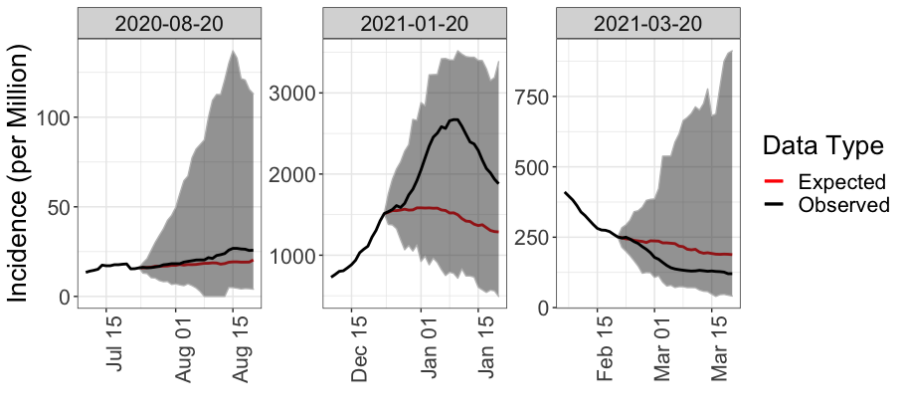
Projected incidence (average and 95% prediction interval) using a 100-nearest neighbor approach, which provides good coverage (observed trajectory lies within the 95% prediction interval). The black line denotes observed incidence rates, while the red denotes the predicted rates, based on an initial period of observation of 14 days; the prediction interval for the predicted incidence over the next 4 weeks is highlighted in dark grey.

Step 1a in our risk modelling procedure was determining the projected incidence, by predicting the number of infectious cases attending a given future event. COVID-19 forecasting is undeniably an involved task, as reflected by its impressive corresponding body of literature (eg, agent-based models or susceptible-exposed-infectious-removed models [39-49]). Predicting the number of new cases per day typically depends on the choice of a specific parameterization (eg, an exponential growth for computing the reproductive number R [50,51]), whose validity is severely hindered by continuous updates to public policies. To alleviate these concerns, we used a nonparametric k-nearest neighbor (kNN) approach. Using all trajectories of the disease incidence across countries and time since the beginning of the pandemic, we computed the k=100 closest trajectories (in terms of the l_2_ loss) on time windows of 2 weeks. The historical trajectories of these kNNs were then used as a “dictionary of observed behaviors” to predict the daily incidence rate in the days leading to the event. We defer to Multimedia Appendix 2 for a more in-depth discussion of this estimation procedure, a description of the parameter selection process, and an evaluation of its performance compared with standard epidemic prediction methods. To briefly summarize, our kNN approach provides a nonparametric, model-agnostic approach to epidemic prediction that is more robust for nonstationarity in public policies than model-based approaches. We show in Multimedia Appendix 2 that these parameters (k=100 neighbors, fitted on trajectories of 14 days) are optimal in allowing an accurate estimation of the trajectory while providing adequate coverage and uncertainty quantification. In fact, we show that, while standard methods fail to provide reliable uncertainty estimates, our kNN methods provide a coverage greater than 95%. Despite coming at the price of wider prediction intervals, our pipeline privileges methods that allow us to correctly estimate the uncertainty in its outputs—thereby more accurately reflecting the state of our knowledge (or lack thereof). Figure 2 presents a comparison of the projected incidences for our 3 dates of interest (August 20, 2020; January 20, 2021; March 20, 2021) for the RAH concert using 2 weeks of fitting and predicting 4 weeks in advance. Note the good coverage provided by our method (the convex hull of the 95% prediction intervals for the projected incidences contains the actual observations). These plots also highlight the importance and variability of the incidence, which varied by orders of magnitude between August 2020 and January 2021.

Step 1b was determining the under-ascertainment bias. The estimated number of new cases based on official incidence data will then need to be corrected for under-ascertainment. The latter refers to the downward bias of the reported prevalence in the population, due for instance, to limited testing capacity, low test sensitivity, or people being unwilling or unable to take a test. To this end, we compared the ratio of the number of deaths over reported cases (translated by 3 weeks) to an expected, age-stratified infection-fatality ratio [52] (see Multimedia Appendix 2 for more details). To highlight the potential importance of this correction step, the ascertainment rate for the United Kingdom was evaluated as over 90% for August 2020 but below 40% for December 2020.

Step 1c was determining the number of infectious participants at the event. Having predicted the background daily incidence rate, we turned to the estimation of the number of infectious participants who will attend the event despite the screening protocols. For an infectious individual to attend the event in spite of the CAPACITY study’s screening protocol, they must (1) have no COVID-19–like symptoms or fail to report them on the morning of the event, (2) receive a (false) negative result during antigen testing D at 2 days prior to the event, and (3) be contagious (rather than simply infected) at the time of the event. We evaluate the joint probability of these events as follows and, for the sake of clarity, refer the reader to Multimedia Appendix 2 for an in-depth explanation of our estimation procedure.

**Figure 3:**
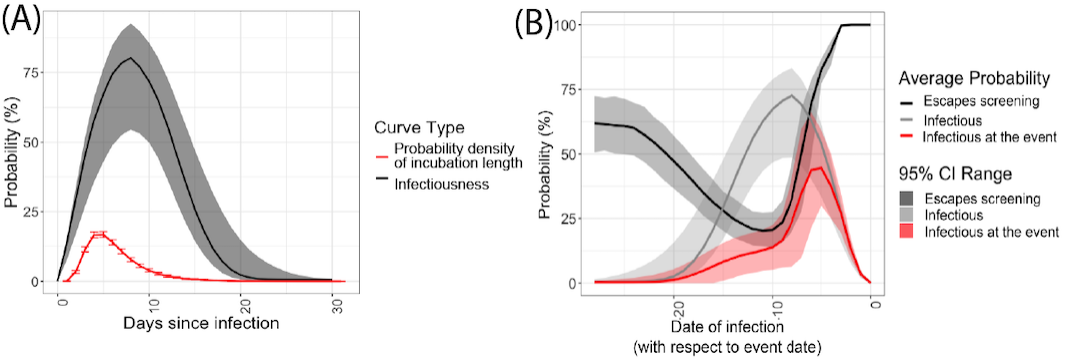
(A) Density of the COVID-19 incubation time and percentage culture positive and (B) probability that an individual is infectious (light grey), that the screening protocol will miss them (black), and that they will be missed and so attend the event (red) as a function of days since infection. The shaded regions denote the uncertainty of this estimate due to the uncertainty on the sensitivity of the test.

Regarding symptom-check failure, one of the main challenges associated with the COVID- 19 crisis is the number of asymptomatic cases—that is, infected individuals who do not express symptoms and are thus unaware of their potential infectiousness. This group includes individuals that are either presymptomatic or completely asymptomatic during the course of their illness—the latter are estimated to represent roughly 25% of all cases [53]. For symptomatic patients, the probability of having symptoms on the day of the event is also a function of time since infection. To account for this temporal dependency, we used estimates of the incubation period (defined as the number of days between infection and symptom onset) from McAloon et al [54] and data on symptom duration from van Kampen et al [55] to estimate the probability for a ticket holder infected k days before the event to exhibit symptoms on the day of the event. A density plot of this probability is displayed in red in Figure 3A.

Regarding antigen test failure, the sensitivity of COVID-19 tests depends heavily on the time since infection—whether these are the gold-standard polymerase chain reaction (PCR) or lateral flow antigen assays [56]. Moreover, studies have shown that lateral flow antigen tests have much lower sensitivity on asymptomatic individuals than symptomatic: In particular, according to a recent Centers for Disease Control and Prevention report [57], rapid antigen testing has 80% sensitivity on symptomatic individuals, but only 40% sensitivity on asymptomatic individuals. Coupling the sensitivity estimates [56,57] with the distribution of the incubation period and estimated percentage of asymptomatic cases [53,54], for each individual infected at day k taking an antigen test D days before the event, the probability of getting through the filtering protocol is thus given by the formula:




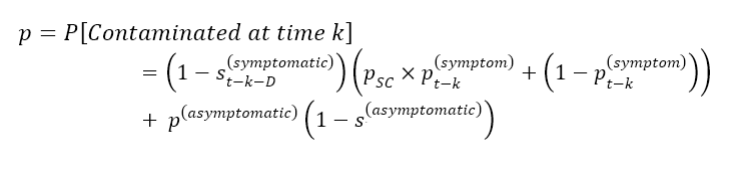




where *s^(symptomatic)^_t–k–D_* and *s^(symptomatic)^* are the sensitivities of the test taken *D* days before the event for a symptomatic participant infected *t–k* days before the event and an asymptomatic individual, respectively. The parameter *p^(symptom)^_t–k_* denotes the probability for a symptomatic individual to exhibit symptoms *t*–*k* days after infection, whereas *p^(symptom)^* is the probability of being asymptomatic. Finally, the variable *p_sc_* denotes the probability of the symptom check failing—namely, that the participant does not want to report their symptoms (see Multimedia Appendix 2 for more details). The curve in black on Figure 3B shows the probability of the failure of the screening protocol as a function of days after infection. The shaded areas denote the uncertainty around this estimate due to the variability of the incubation time.

The infectiousness of the participants—that is, the propensity of an infected ticket holder to contaminate others—is a function of time since infection. In order to estimate this relationship, we build upon the existing literature studying the link between reverse-transcription PCR thresholds and cultivable virus [58,59]. The percentage of culturable viral material in the sample can indeed be used as a proxy for infectiousness. Using the estimated percentages of viable samples [58,59] as a function of time since symptom onset, compounded with distribution of the incubation period duration [54], we computed an estimate of the infectiousness as a function of time since infection (black curve in Figure 3A). A more complete description of this estimation procedure is presented in Multimedia Appendix 2. The results are presented in Figure 3B. The red line in Figure 3B shows the resulting probability for an infectious ticket holder to pass through the screening protocol and be allowed into the event. Note that ticket holders that have been infected 5 days before the event are the most likely to be infectious and let in the venue on the day of the event.

Step 1d was determining the number of participants at risk. Finally, the last quantity that we needed to infer before getting into the specifics of the transmission mechanisms was the number of participants at risk of being infected who present at the event. This requires a knowledge of the participants’ COVID-19 susceptibility status (ie, has the participant already had COVID-19 in the previous year, or has the participant been vaccinated?) While previous history could be imputed through additional questions (eg, previous positive test for COVID-19 and symptoms combined in a model such as in [60]), for the sake of simplicity, we only considered the vaccination status of the participants—thus leaving out the proportion of the population that had COVID-19 but was not yet vaccinated. This induces a risk estimate that is biased upward and is thus more conservative. We imputed missing data (cases where the participants have not filled in their vaccination status) using linear regression, expressing vaccination rate as a function of time. This assumes that vaccinations are operating at capacity (see Multimedia Appendix 2 for a longer discussion on the reasons for this approximation and further ways of improving this model). Having imputed the rate of new vaccinations π_s,_ *s*=1…*t* days leading to the event, we turned to the estimation of the number of individuals that are likely to be susceptible. Recent reports indicate that vaccine-acquired immunity is a function of both time since vaccination and number of doses [61]. To compute the effective number of participants at risk in the event, we used a compound Poisson distribution: On each day *s* in the weeks leading to the event, the number *X* of new participants vaccinated (having either their first or second dose) is expressed as a Poisson(π^(^*^dose j^*^)^), where *j* ∈ {1,2}. Each of these newly vaccinated individuals then has a probability *ρ*^(dose j)^ of being immune, depending on the date and dose *j* that they have received. The resulting number of immune people Z attending the event can thus be modelled as:




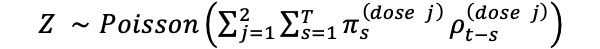




We discuss in Multimedia Appendix 2 how this estimation can easily be modified as the vaccination rates increase and the Poisson approximation becomes no longer valid.

#### Royal Albert Hall Example

For the RAH example, we present a comparison of each quantity for 3 different dates (see Table 2). Of note is that the screening safety protocol is effective in more than 60% of cases, that when combined with the expected infectiousness of participants and self-reporting of COVID-19–like symptoms, implies that 95% of infected cases are removed. We also note that prevalence is very important in determining the number of infectious cases at the event—thereby highlighting the importance of a context-aware risk calculator. The combined effect of the screening protocol and the natural time-dependent infectiousness of infected ticket holders means that the number of infectious participants at the event is likely to be very low (~ of the order of tens in times of extremely high prevalence).

**Table 2:** Comparison of the efficiency of the screening protocol and the number of infectious participants at the event by date.

Measurement	August 20, 2020	January 20, 2021	March 20, 2021^a^
Projected incidence (in 1,000,000)	20	1286	188
Number of infected participants	3.6	299.3	50.2
Number of infectious participants at the event	0.22	7.96	2.00
Percentage of caught cases, %	94	97	96
Number of susceptible participants	4996.4	4700.7	3860.4

^a^Vaccination rates started to account for a substantial proportion of the British public, so that the sum of the number of susceptible participants and the number of infected participants does not equate 5000.

### Step 2: Modelling Transmission Dynamics

Having estimated the number of infectious participants at the event, the second major component of our model consists of estimating the number of transmission events during the event itself.

#### Identification of Transmission Mechanisms

More than a year after the start of the epidemic, the precise mechanisms by which COVID-19 is transmitted are still unclear. Aside from direct physical contact, experts continue to debate the significance of the following 2 main routes of infection: droplet transmission and airborne transmission.

In the scenario of droplet transmission, transmission happens through the inhalation of droplets (particles of 5-10 µm in diameter [62]) and typically occurs when a person is in close proximity (within 1 meter) of someone who has respiratory symptoms (eg, coughing or sneezing).

Increasing concerns around airborne transmission have been raised by a number of experts over the past few months [63,64]. Airborne transmission refers to the presence of the virus within droplet nuclei remaining in the air for long periods of time and with the potential to travel long distances [63] and penetrate more deeply in respiratory tracts. Airborne transmission has been estimated to be nearly 19 times more likely indoors than outdoors [65]. In the context of large public events, this transmission route thus has more diffusive power and hence could explain several super-spreader events (SSEs) [6], making it a major cause for concern [2,63,66-72].

While droplet emission is undeniably a source of concern and a major source of transmission, simple safety precautions such as mask wearing have been shown to efficiently control this transmission source [73,74]: It is estimated that face masks can block 80% of exhaled droplets and reduce inhaled droplets by up to 50% and so, on average, reduce the transmission probability by 70% [73]. Conversely, the evidence concerning the efficiency of standard protective equipment in filtering aerosol droplets varies widely across studies probably due to “variation in experimental design and particle sizes analyzed” [73]. Airborne transmission in indoor settings can thus represent one of the main risk factors in live events, which we focus on modelling using the aerosol model proposed by Jimenez and collaborators [21,69,75]. This aerosol transmission model is currently one of the only COVID-19 transmission models that provide enough granularity to quantify the risk associated with an event. This recognized model has been used several times in the literature over the course of the pandemic [76], including to allow in-class teaching at the University of Illinois at Chicago [70]. Based on the Wells-Riley model [77-79], this estimator calibrates the quanta to known transmission events and considers important factors to compute a risk estimate, including event-specific (eg, number of people, local prevalence) and venue-specific (ventilation rate, size of the venue, UV exposure) variables. This Wells-Riley–based model relies on the evaluation of 3 quantities: (1) the quanta exhalation rate, which is contingent on the activity performed and the number of infectious participants*;* (2) quanta concentration, which is a function of the volume of the space, the room ventilation rate, and the quanta exhalation rate; and (3) quanta inhalation rate, which is a function of the quanta concentration and breathing rate associated with the activity performed. The probability for each susceptible individual to be infected can then be written as *p_infection_*=1 – *e^–qinhalation^*. See Multimedia Appendix 3 for more details.

#### Modelling the Uncertainty of the Model

To estimate the uncertainty associated with this model, we used Monte-Carlo simulations. We simulated random input parameters (number of infectious and susceptible individuals) using the distributions and uncertainty estimates discussed in the previous section. In order to model the uncertainty associated with the aerosol transmission model, we added a sampling step at the end of the Jimenez and Peng pipeline. This allowed us to account for individual variations in infectious participants’ ability to spread the disease and to remain consistent with the extensive literature on the heavy-tailed Pareto nature of COVID-19 transmission and superspreading [24-27]. For each infected participant, we sampled the number of quanta that they exhale using a Pareto distribution with shape θ = 1.16 and rate η = θ/(θ – 1)*q*^exhalation^. This produces a distribution centered around *q*^exhalation^ but skewed to the right and heavy-tailed—thereby modelling the heterogeneity in infected participants’ ability to spread. This choice of parameters allowed us to abide by the Pareto principle, according to which 80% of transmissions are due to 20% of those infected. In accordance with the uniform mixing assumption of the aerosol transmission models, susceptible participants then all inhale a quanta concentration that is a function of the sum of the exhaled quanta: All have an identical probability of becoming infected. In mathematical terms, infections are thus simulated using a binomial distribution such that *n_infected_* ~ Binomial(*n_susceptible_*, 1 – *e*^–qinhaled^). We discuss the limitations of this approach and its assumptions in the discussion section of this paper.

The code for the model can be found online on the authors’ Github [80].

## Results

### Step 3: Comparison With the Null Model

To quantify the effect of the event, it is necessary to put it in context of the background rate of infections: Even if the participants had not been to the event, they could have been infected elsewhere. In this null model, the number of infections is binomially distributed, such that the number infections Y is Y ∼ Binom(n_susceptible_, π).

We present the results for the RAH example in Table 2. This table shows in grey the values of the different quantiles of this distribution. We note the skewed distribution that we obtain is expected given the modelling of the uncertainty around inhalation rate. If the event did not occur, then on each respective date, there would be an expected community transmission of 0.07 (95% prediction interval: 0-1), 2.5 (95% prediction interval: 0-7), and 0.63 (95% prediction interval: 0-3) events on August 20, 2020, January 20, 2021, and March 20, 2021, respectively. However, with the event taking place on these dates and calculating the expected number of infectious individuals, susceptible individuals, and transmission dynamics within the venue, the distribution of the number of transmission events would in general widen to 0.06 (0-1), 2.38 (0-19), and 0.67 (0-6) in that same order. In this case, it is important to note the similarity in mean transmission between the “event” and “no event” scenarios and their substantial deviation in the tails. This highlights the importance of modelling the distribution of the risk and highlighting its substantial heavy tails, rather than providing point estimates.

It is likely, although not inevitable, that the event will have an impact on the transmission and increase it irrespective of the level of the prevalence. However, for low levels of prevalence and higher vaccination rates, this substantially decreases. Having computed the number of expected transmission events, we can then compute several complementary metrics of interest including, for example, the secondary attack rate (SAR)—that is, the number of COVID-19 cases in the participants’ community in both the null and event models. SAR can be calculated from the predicted reproductive rate (R) in the regions where the ticket holders dwell. In the United Kingdom, R rates are updated on a weekly basis at regional levels (eg, East Midlands, London) and available from the Office for National Statistics or can be derived from the kNN modelling previously described. An opportunity for further research would be to estimate SAR within households by gathering contextual data from ticket holders. Equally, estimates of hospitalizations and deaths might be possible based on individual characteristics and comorbidities; however, this is beyond the scope of the current article.

### Evaluating the Effectiveness of the Screening Protocol

**Figure 4:**
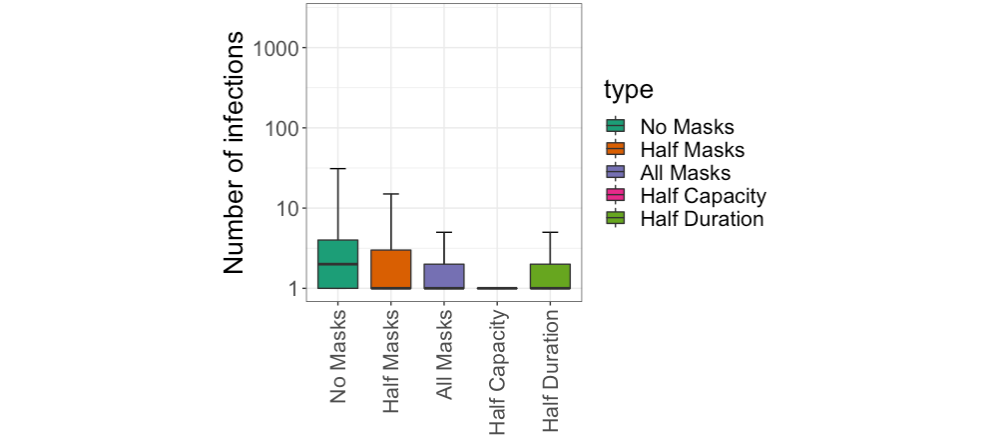
Boxplots showing the distribution of the number of infections across different scenarios, for our Royal Albert Hall event held on March 20, 2021: Where variables are not mentioned, the number of attendees is 5000, the duration is 3 hours, and the proportion of attendees wearing masks is 100%.

This risk modelling pipeline also allows comparison of different protocols and situations. For example, this pipeline highlights (1) the importance of event duration (the longer the dwell time at the event, the more at risk the participants) and (2) the importance of wearing masks. Table 3 quantifies the outcomes of holding the event on our 3 dates, assuming that either 0%, 50%, or 100% of participants are wearing masks or varying parameters such as the density or length of the concert. Figure 4 completes that analysis by providing a visual representation of the effect of these parameters on the distribution of the number of infections. The distributional nature of these results is essential in highlighting nuances between scenarios: While holding an event at half capacity or for half the duration produces average transmission risks that are roughly similar, holding the event at half capacity seems to more substantially reduce the effect of the event in the tails of the distribution.

**Table 3:** Effect of different input parameters on the quantiles of the number of infections for an event at the Royal Albert Hall across all 3 dates.

Event	August 20, 2020, median, mean (99% CI)	January 20, 2021, median, mean (99% CI)	March 20, 2021, median, mean (99% CI)
No mask wearing, 3 hours, n=5000	0, 0.3 (0-4)	5, 9.9 (0-76)	1, 2.4 (0-21)
50% mask wearing, 3 hours, n=5000	0, 0.2 (0-3)	3, 5.5 (0-40)	1, 1.3 (0-13)
100% mask wearing, 3 hours, n=5000	0, 0.1 (0-1)	1, 2.4 (0-19)	0, 0.7 (0-6)
100% mask wearing, 1.5 hours, n=5000	0, 0.04 (0-1)	0, 1.4 (0-10)	0, 0.4 (0-3)
100% mask wearing, 3 hours, n=2500	0, 0.2 (0-1)	0, 0.9 (0-8)	0, 0.2 (0-3)

In addition to the aggregated risk that a live event presents, individual risk of transmission can be estimated and can be communicated to ticket holders so that they can gauge whether the risk of attending the event outweighs their desire to attend. For the first person to purchase a ticket, risk of transmission will be calculated based on their own immunity status (eg, vaccination, regional prevalence) and a synthetic population based on national prevalence at that time. As more bookings are assigned to ticket holders, the reliance on the synthetic population decreases as understanding of the number of susceptible and potentially infectious individuals attending the event increases. Therefore, the confidence in the risk score increases as the event draws closer and as the proportion of tickets sold increases. This can be reflected in the updated risk scores provided to ticket holders as the event approaches. The individual risk scores can be modified based on alternative scenarios imputed into the risk algorithm. For example, for an individual not yet vaccinated, their risk could be also presented as if they had been vaccinated, offering an opportunity for the individual to appreciate how vaccination could have modified their risk. Such an approach could form the basis for behavior change interventional studies for promoting health literacy and tackling vaccine hesitancy (see Multimedia Appendix 1). By working in partnership with the live events organizer, individuals that chose to opt out can be reimbursed without delay and the ticket re-sold.

## Discussion

The modelling we propose is based on prevalence estimates and screening protocols to calculate the number of infectious and susceptible individuals attending the event as well as transmission dynamics at the venue to predict the number of new infections. Our paper demonstrates the value of estimating attack rates from live events so that they can be appropriately managed. We also demonstrate how individual ticket holders can receive personalized risk scores for contracting COVID-19 at the event, which would, for the first time, enable genuine informed consent to be obtained. Although this methodology provides clear benefit to event organizers, local public health authorities, and individual ticket holders, our approach is based on several assumptions that group in 2 categories: modelling assumptions and parameter sensitivity.

### Modelling Assumptions

As they combine data and tools from different sources, the computations in our pipeline rely on assumptions at 3 main levels: predicting COVID-19 prevalence, assessing the efficiency of the screening protocol, and transmission at the event.

#### Predicting COVID-19 Prevalence

To predict future COVID-19 incidence, we chose a kNN approach as it yields a more robust prediction and better uncertainty quantification than most existing parametric methods. One of the downsides of this approach is that it might not generalize very well to entirely novel behaviors or viral variants—in which case well-parameterized methods may outperform our approach as knowledge of transmission, vaccination, and other relevant model parameters continues to improve. While prevalence predictions are important for event planners and attendees alike, on the day of the event, the more important metric is whether official case rates reflect actual cases (ie, the ascertainment rate). Historically, this rate has been low due to limited testing facilities, and our method to determine ascertainment using cases, deaths, and infection-fatality rates reflects this, but also indicates that ascertainment may exceed 100% in times of widespread testing and low prevalence. It was beyond the scope of this paper to further investigate ascertainment, but we expect that future research will clarify the impact of different test types, their false negative and positive rates, and their frequency of use in determining the ascertainment rate.

#### Assessing the Efficiency of the Screening Protocol

Our modelling framework assumes that events will screen participants with COVID-19 tests, such as virtually witnessed lateral flow antigen tests. Assessing the efficiency of this screening step requires the estimation of (1) the sensitivity of the test, (2) the probability of having symptoms, and (3) the probability of being infectious—all of these quantities being a function of days since infection. Our estimation of each of these quantities is based on published data—with the exception of the probability of symptom check failure (ie, the probability that a participant lies about their symptoms to get in). By default, we select this probability to be 50%, a choice that will be improved upon as the CAPACITY and other similar studies gather behavioral data. However, as shown in Multimedia Appendix 4, this factor has a relatively minor impact on the outcome of the model compared with the uncertainty of the other inputs. Of potentially greater concern is our assumption that the probability of testing negative 2 days before the event is independent (conditionally on time since infection) of a participant’s infectiousness during the event. A potential avenue for improvement could consist of determining both test sensitivity and infectiousness as a function of viral load and estimating the joint probability of the viral load 2 days apart. However, the data required for this approach are—to the best of our knowledge—still lacking and given the variability of the viral load or PCR cycle threshold behavior, this conditional independence assumption seemed a reasonable first-order approximation.

#### Transmission at the Event

The airborne transmission model that we use relies on a homogeneous (well-mixed) air hypothesis for an indoor environment. While several other models have been proposed (either breaking the room into compartments or using a distance index) to counter this hypothesis, we highlight (following the discussion by Jimenez and Peng [75]) that this is a first-order approximation: Some participants will have more risk and others less, so that at low quanta concentration, this effect will be averaged out. At very high concentration, the model will likely underestimate the number of infections, but given the efficiency of the screening protocol and density limitations, we do not expect this scenario to be common. Moreover, while this model was originally developed for indoor transmission, its application to an outdoor setting—where the ventilation rate can be considered infinite and transmission is more likely to occur through droplets rather than aerosolized particles—can nonetheless provide a conservative estimate of the risk. We are however currently working on developing a better model for outdoor transmission, relying on a modelling of droplet transmission in crowd bottlenecks. We leave the detail of this separate transmission model to future work. Finally, we note that our model is not tied to any specific transmission mechanism, and as our knowledge of COVID-19 transmission improves, we can refine and supplant the transmission dynamics with a superior alternative or another model that is deemed more suitable.

### Parameter Sensitivity

While we try to limit the number of input parameters in our pipeline, the sensitivity of the estimates to these inputs (namely, the mask efficiency and population of interest) has to be studied. We refer the reader to Multimedia Appendix 4 for a quantitative sensitivity analysis and highlight our conclusions here. In terms of the model parameters, the greatest unknown consists in determining the efficiency of masks and protective equipment—the latter having been shown to vary depending on the mask type and activity. However, we hope to make use of the growing body of literature on the topic to update and refine this important factor. Second, our prediction framework assumes that participants at the event have the same probability of infection and vaccination as their regional average. However, this might not be the case as participation in the event may be an incentive to get vaccinated or conversely might select for less cautious subpopulations. The importance of this sampling frame assumption nonetheless decreases as participants’ vaccination status and behavioral data from the CAPACITY study will result in more precise estimates.

### Model Validation

Finally, one of the main current hurdles for developing risk estimators lies in the absence of quality data to validate and benchmark different transmission models—thereby making the task of validating our transmission pipeline a rather daunting task. Indeed, while we can (and have, see Multimedia Appendix 2) check the accuracy of the vaccination and prevalence estimation step, the validation of the transmission model itself is inherently difficult: There are no, or very few, available datasets on COVID-19 spread following live events or rigorous accounts of SSEs, nor are there any statistics on how likely SSEs are. As such, the majority of SSEs that are documented currently (1) are generally not detailed enough to untangle the huge variability in context (eg, outdoors vs indoors, activity performed, background prevalence) and (2) suffer from selection bias—and might not be reflective of the general distribution of live events. To make up for the current lack of testing data, we resort here to the following 3 strategies: model checking, model validation on (scarce) existing data, and prospective data gathering.

For model checking, we begin by validating the behavior of our model estimates on documented SSEs [81]—that is, we confirm that the model outputs (1) present similar tail behavior as these documented SSEs and (2) are predicted as outlier SSE events by our model.

For model validation on (scarce) existing data, we also consider 2 documented live indoor concert events [82,83] and use the event parameters as well as the documented transmission statistics to verify that these numbers fall within the realm of feasible outcomes.

For prospective data gathering, finally, to overcome the lack of available data, we propose using the RShiny app [38] as a data collection platform and encourage users (event organizers and participants alike) who use the app to record their event in our dataset by filling in a survey [84]. This paves the way for a larger-scale and more detailed record of transmission events at large gatherings, as well as a more precise modelling of transmission dynamics.

This validation and model assessment step is further described in Multimedia Appendix 5.

### Conclusion

A nuanced, data-driven system is required to assess risk at each event informed by the characteristics of all ticket holders and the background risk of transmission concurrent to the event, so that proportionate and specific action can be taken by event organizers and public health authorities. We have detailed our attempt to create such a system and have outlined its predictions and limitations. Our end-to-end risk model is provided in the form of an RShiny interface. At times of high prevalence, this type of system will ensure events likely to increase transmission can be halted. At times of low prevalence, this will ensure events can potentially continue to operate. Learning to live with SARS-CoV-2 will be about implementing systems that support hyperlocal, data-driven decisions so that far-reaching and highly damaging sector-specific lockdowns can be avoided as much as possible.

## Appendix

Multimedia Appendix 1 Prediction.

Multimedia Appendix 2 Model and assumptions of the Jimenez aerosol transmission model.

Multimedia Appendix 3 Sensitivity analysis.

Multimedia Appendix 4 Risk communication.

Multimedia Appendix 5 Model validation.

## Abbreviations

ARC: Applied Research Collaboration
kNN: k-nearest neighbor
NIHR: National Institute for Health Research
PCR: polymerase chain reaction
RAH: Royal Albert Hall
SAR: secondary attack rate
SSE: super-spreader event
