# Supplementary material for: Predicting COVID-19 Transmission to Inform the Management of Mass Events: Model-Based Approach
Source: JMIR Public Health Surveill. 2021 Dec 1;7(12):e30648. doi: 10.2196/30648 (PMC8638785; doi:10.2196/30648)
Supplement: Multimedia Appendix 1 [file publichealth_v7i12e30648_app1.docx]

# Appendix A: Prediction

As explained in the introduction, the aim of this paper is to develop a context-aware risk model. To be informative, the estimates of the risk that the model outputs must informed by (a) the prevalence at the time of the event, (b) the ticket holders’ vaccination status, and (c) the screening protocol employed by the event management to reduce transmission risk. In this setting, the CAPACITY protocol serves as a case in point and a motivation to our paper. Central to the study is the estimation of the risk associated with the event on two different horizons:

***Horizon 1: Weeks prior to the event.*** In this setting, the goal is to predict ahead of time the risk associated with the event, so as to help organizers and participants alike to plan ahead and decide whether or not they deem the risk associated with the event acceptable. *This step requires the prediction of both the prevalence of the disease and the vaccination status of the crowd several weeks in advance.*

***Horizon 2: A few days before the event.*** The purpose of the risk estimation is to evaluate – with more certainty – the admissibility of the risk associated with the event. In this step, *the algorithm can rely on ticket holders’ reported vaccination status, as well as the most recent incidence rates to compute the risk*. This allows in particular to reduce the uncertainty in the prevalence rate substantially, and circumvents the problem of imputing prevalence and vaccination status.

Thus, ***whilst not crucial for Horizon 2 (in the last few days leading up to the event), the prediction of the crowd’s vaccination status as well as the incidence rate are major components of Horizon 1*** – thereby calling for prediction methods that both provide accurate estimates and a correct evaluation of their associated uncertainty. In this appendix, we focus on providing more details into the different predictive components that we use to estimate the risk ahead of time (Horizon 1). These consist of the following three main steps, which we subsequently describe in greater details:

- - **Step a:** The prediction of the number of newly infected individuals who are ticket holders.
  - **Step b:** The prediction of the number of infected participants that will escape the screening protocol.
  - **Step c:** The prediction of the number of vulnerable ticket holders at the time of the event (i.e., participants that are not immune).

**Step a. Prediction of new cases through a k-Nearest Neighbour (k-NN) Approach.** The first step in our pipeline consists of the estimation of the daily incidence rate in the days leading to the event, which, in the main text of this paper, we suggested solving using a *k*-nearest neighbour approach with $k=100$. Specifically, we consider the trajectories of the daily incidence rate (per million) over all countries, and across different points of the epidemic on intervals of $D_{train}=14$ days. This creates a “dictionary” of over 30,000 observed epidemic-kinetics behaviours. We then determine the $l_{2}$ distance between the last $D_{train}=14$ days of the trajectory of interest (that is, the trajectory for which we want to predict the next $D_{predict}$ days) and all fourteen-day historical trajectories in our dictionary. We then retain the closest $k=100$ neighbours (corresponding to the smallest $l_{2}$ distance), and translate them appropriately so that their last day of fitting matches the last value recorded in our trajectory of interest. We summarize these $k=100$ trajectories to get a prediction and prediction interval for our trajectory of interest: we use the mean, 2^th^ and 98^th^  quantiles of the historical trajectories of the 100 closest neighbours over $D_{predict}$ days to predict the expected incidence rate and provide a prediction interval for the next $D_{predict}$ days. The procedure is summarized in Algorithm 1.

The choice of $k=100$ neighbours allows us to have sufficient information to evaluate the percentiles of the distribution of the prediction, whilst retaining a sufficient amount of similarity with the original trajectory. The choice of 14 days is motivated by the fact that we need sufficient data to find trajectories with similar behaviours, whilst remaining sufficiently local for the comparison to be valid: epidemic kinetics change from month to month, or even week to week, so the similarity in epidemic kinetics might only hold for a limited time frame. We provide more details on the selection of these parameters, as well as compare this method against more commonly used benchmarks in the subsequent paragraph.

| **Data:** Previous Incidence data $Y\in\mathbb{R}^{n\times T}$ ;  Choice of k=100;  Input vector $X\in\mathbb{R}^{p_{train}}$ with *p*_train_ the length of the training period;  *p*_pred_ the desired length of prediction period;  **Result:** k-Nearest Neighbors sample trajectories of length *p*_pred_, average prediction and uncertainty estimates Step 1: Compute the distance matrix: **for** *i in 1:n* **do**  **for** *t in 1:T by 14* **do**  Compute distance: $D_{\left\lfloor t/14 \right\rfloor+1,i}=\text{distance}\left( X,Y_{\left( t-p_{train}+1 \right):t} \right)$; end **end**  **Step 2: Extract k nearest neighbours**:  $\mathcal{I,J}$= Row and Column Indices of the k-smallest elements of D; Step 3: Recenter (translate) the prediction on start date to start the predicted trajectory: $\hat{Y}\left[ 1:p_{pred} \right]=\text{Mean}(Y\left[ \mathcal{I,J:}\left( \mathcal{J+}p_{pred} \right) \right]+X\left[ 1 \right]-Y\mathcal{[I,J]}$; |
| --- |

**Algorithm 1:** Prediction of the epidemic curve using k-NN

*Motivation for the k-NN approach.* COVID-19 prediction is undoubtedly an involved task — as denoted by the impressive amount of literature published on the topic [1-11]. Yet, as emphasized in the main text, many of these methods rely on a parametrization of the problem (Exponential growth, SEIR model, etc. [12,13]) which require input parameters (e.g., the reproductive number) that are both unknown and non-stationary. Indeed, as the number of cases rises, policy makers are bound to adapt their policies to limit the spread of the virus. Reciprocally, as community prevalence levels drop, strict lockdown measures and stay-at-home orders are bound to be lifted — thus impacting the transmission modalities and likelihood of propagation of the virus. ***Incorporating both uncertainty and non-stationarity in COVID predictions is thus a challenging task***. Our solution to this problem relies on using a fully non-parametric, ***model-agnostic k-Nearest Neighbour (k-NN) approach:*** this approach essentially predicts the behaviour of the incidence rate (normalized per million people) using historical data. We argue that such a non-parametric approach to fit growth epidemic curves has the potential to be more accurate than other parametric models that (a) rely on a specific sets of assumptions (ie, compartmental models), (b) are only valid for a limited amount of time (e.g, Exponential Growth models), and (c) assume a stationary, identical regime of growth/ public policy throughout their predictions. By contrast, historical trajectories contain information both on the reproductive number and propensity of the epidemic to grow, but also on policy decisions made as a result of rising (or declining) prevalence numbers — thus making them an appealing non-parametric candidate for non -stationary incidence modelling.

*Selection of the k-NN parameters.* While k-NN is a non-parametric, model agnostic approach that does not make any assumptions on epidemic kinetics nor effects of public policy attempting to curb the spread, it requires nonetheless the selection of three critical parameters: (1) The similarity function to determine the nearest neighbours, (2) the number of nearest neighbours $k$, as well as (3) the number of days $D_{train}$ used for training. In the previous two paragraphs, we detailed the k-NN procedure providing the values of the parameters that we use in our final algorithm. This selection stems in fact from the results of a grid-search over candidate values for both $k$, $D_{train}$, and the similarity function:

- **Similarity function:** The choice of the distance function that determines the resemblance between two incidence trajectories is a crucial component of the algorithm. We compare the use of four similarity functions:
  - *Simple MSE.* In this case, denoting $X_{i},X_{j}\in R^{D_{\mathrm{train}}}$ two incidence trajectories, their similarity is simply given by: $s_{ij}^{MSE} = \frac{1}{D_{train},}\sum_{t=1}^{D_{\mathrm{train}}} \left( X_{it} - X_{jt} \right)^{2}$.We refer to this distance as the “Mean Squared-Error” (MSE).
  - *Weighted MSE.* To place more weight on matching the most recent observations in the trajectories, we consider a version of our MSE metric in which the weights of observations are inversely proportional to the time since the origin of prediction (i.e., for each day $k\in\left[ 1,D_{train} \right], w_{k}=\frac{1}{D_{train} + 1-k}$), so that: $s_{ij}^{W-MSE} = \sum_{t=1}^{D_{\mathrm{train}}} \frac{1}{D_{train}+1-t} \left( X_{it} - X_{jt} \right)^{2}$.
    This weighting puts more emphasis on recent observations, so that trajectories that share a higher degree of similarity in their recent behaviours will be favoured by the algorithm. We will denote this method by “Weighted Mean Square Error”.
  - *Correlation.* Another way of measuring similarities between distributions consists in using a correlation measure between trajectories, so that the distance between two trajectories is simply: $s_{ij}^{corr} = 1-\sum_{t=1}^{D_{\mathrm{train}}} \frac{X_{it}-\bar{X_{i}}}{\left| \left| X_{i} \right| \right|} \frac{X_{jt}-\bar{X_{j}}}{\left| \left| X_{j} \right| \right|}=1-corr\left( X_{i},X_{j} \right)$. Compared to the MSE metric, this measure only considers the shape and direction of increase, but does not consider information in the actual value of the incidence itself: contrary to the MSE, this measure encourages matching rates of increase, regardless of the incidence rate in one curve is above 500 in one curve, and 10 in the other – vastly different COVID regimes, that can potentially yield very different public policy measures as a response.
  - *Weighted Correlation.* This distance is an extension of the correlation-based distance, which gives more weight to recent observations (in a similar manner than for the “Weighted- MSE”). The distance between two trajectories is simply: $s_{ij}^{w-corr} = 1-\sum_{t=1}^{D_{\mathrm{train}}} \frac{1}{D_{\mathrm{train}}+1-t} \frac{X_{it}-\bar{X_{i}}}{\left| \left| X_{i} \right| \right|} \frac{X_{jt}-\bar{X_{j}}}{\left| \left| X_{j} \right| \right|}$.
- **Determining the k in k-NN:** The estimation of the percentiles for the distribution requires us to have at least 100 nearest neighbours. However, the choice of the number of neighbours is subject to the classical “bias versus variance” trade-off: as $k$ increases, the bias of the model also increases, as we might be using trajectories that are too different to be useful. In our grid-search, we thus use {100, 200, 300, 500} as candidate numbers of curves to do our predictions.
- **Determining the length of the training period**: Similarly, the length of the training set (that is, the number of days that we use to find “nearest historical trajectories”) is also “subject to a bias versus variance” tradeoff: epidemic kinetics are rapidly varying, and change from month to month, or even week to week. As such, the selection of the appropriate length for our fitting window must arbitrate between being short enough to capture recent epidemic developments, but sufficiently long to correctly capture similar behaviours. To determine this parameter, we also use a grid-search approach, allowing the training window to vary between {7, 14, 21, 28} days.

*Benchmarking of the k-NN approach.* To provide more substantial ground for our proposed k-NN approach, we studied its performance compared to more traditional benchmarks using exponential growth model and Attack rate models [12, 13], commonly used in the literature and implemented in the R package R0 [14].

*Grid-search and benchmark experiments.* We compute the performance of each method and vary the similarity, k, and length of training period to establish the optimal set of parameters. To summarize and be fully explicit, our comparison thus focuses on comparing:

- - The Attack Rate method [12], using the R packages R0 [14], Incidence [15] and Projections [16],
  - The Exponential Growth model [13] (using the same R packages),
  - Our k-NN method with MSE distance, W-MSE, correlation or W-Correlation distances.
  - Our k-NN method with an MSE distance, but computing the prediction using the median of the k-nearest neighbours rather than the mean.

To compare these methods, we use data from "Our World in Data": for each country, we evaluate the performance of each method for predictions between June 1st 2020 to July 1st 2021. Every two or three months (depending on the rapidity of the spread: June 1^st^ 2020, September 1^st^, November 1^st^, January 1^st^ 2021, March 1^st^, July 1st ), we recreate a prediction scenario. We use {7, 14, 21, 28} weeks of observations for training, and predict the daily incidence for the next four weeks (28 days). The purpose of this experiment is to assess and compare across methods (a) the methods’ prediction accuracy, and (b) the coverage (percentage of times that the confidence interval covers the true observations), in order to establish which method allows to correctly estimate the uncertainty and risk. Figure 6 presents a comparison of statistics of the Weighted Root Mean Square Error (W-RMSE, defined as $\sqrt{\sum_{t=1}^{D_{pred}} \frac{1}{D_{pred}+1-t} {\left( \hat{X}_{it} - X_{it}^{obs} \right)^{2}}}$), averaged over all countries and all dates (top panel) and for the 95^th^ percentile of the W-RMSE, showing how robust and well-behaved these predictions are (bottom panel). From these graphs, we can clearly see the bias versus variance effect trade-off effect in the number of curves: the accuracy decreases (as denoted by an increase in RMSE) as the number of k-nearest neighbour curves used for the prediction increases. Similarly, predictions are less accurate for longer fitting windows. Overall, we observe the superior performance of the MSE method – that is, when using the MSE as a similarity score, and using the median of k nearest neighbours (instead of the mean) to do the prediction. In particular, a choice of k=100 for a training window of 14 days seems to provide the best results (best mean and 95^th^ quantile) – see Figure 6 and Table 2.

| **Method** | **Training Window Length** | **Number of Closest Neighbours** | **Mean W-RMSE** | **Median W-RMSE** | **95^th^ Quantile of W-RMSE** | **Mean Width of the Prediction Interval** |
| --- | --- | --- | --- | --- | --- | --- |
| Median-MSE | 14 | 200 | 41.1 | **8.67** | 202.0 |  |
| W-MSE | 14 | 100 | 39.9 | 9.53 | 186.7 |  |
| MSE | 14 | 100 | **39.7** | 9.57 | **183.3** |  |
| Correlation | 7 | 100 | 47.3 | 9.71 | 244.0 |  |
| W-Correlation | 7 | 500 | 48.2 | 9.83 | 250.4 |  |
| Attack Rate | 21 |  | 55.2 | 11.70 | 264.4 |  |
| Exponential Growth | 28 |  | 67.8 | 20.47 | 1815 |  |

Table 2: Summary for the goodness of fit tests on the test data. Results correspond to the mean, median and 95^th^ quantile of the W-RMSE over all dates and all countries in the test data. We show the best performing model from each category. We note the clear superiority of the kNN neighbour method over the Attack Rate and Exponential Growth model: the MSE based classification model achieves a reduction in W-RMSE of respectively 28% and 41%.


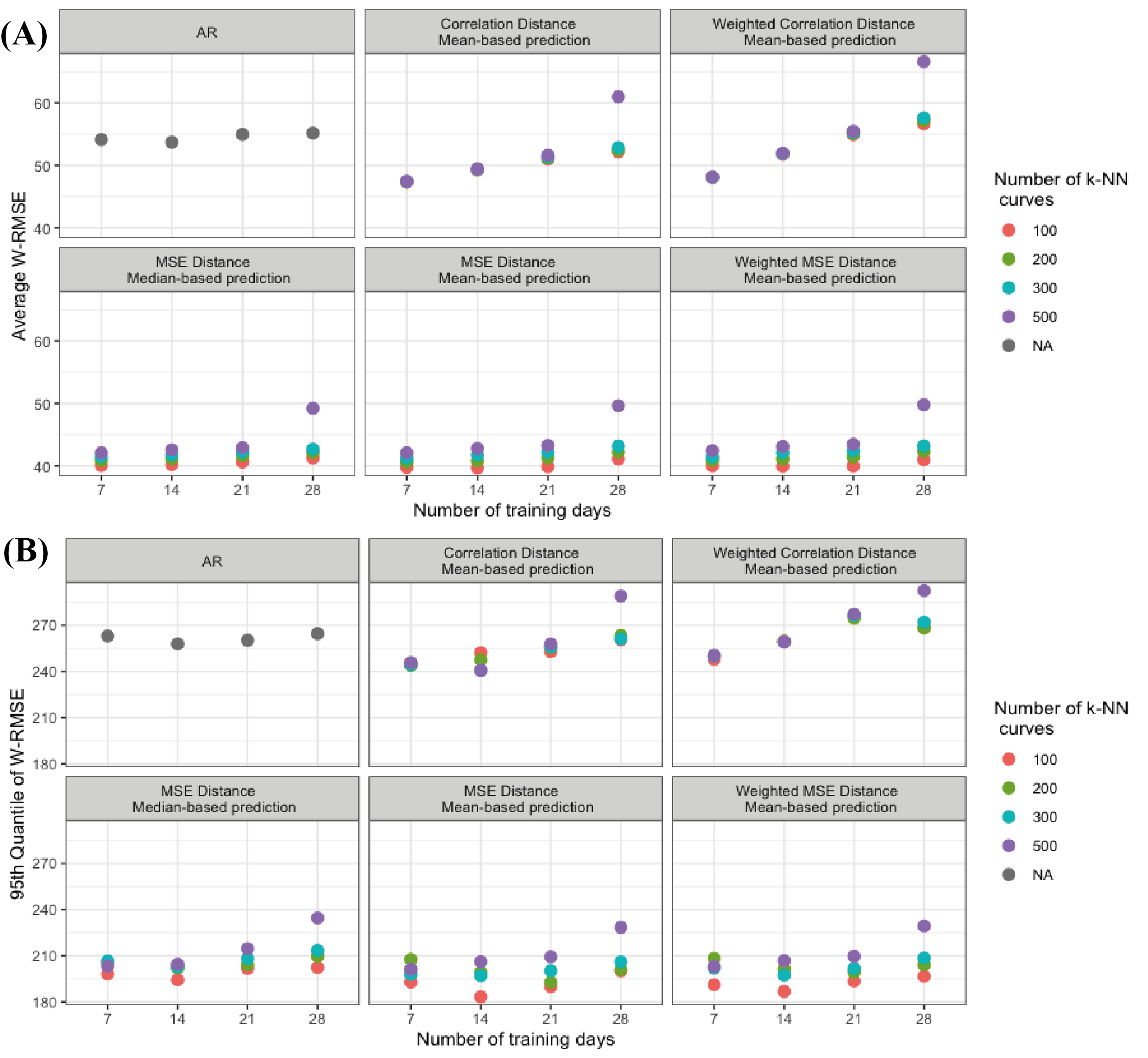


Figure 6:Weighted Root Mean Square Error for the different methods (mean and 95^th^ quantile). We note the robustness of the mean and median based-MSE to the fitting window length.

The results across countries and periods are provided in Figure 6, displayed for a subset of countries on Figure 7 and the coverage is shown in Figure 8. We see that the k-NN method achieves comparable weighted Root Mean Square Error (W-RMSE) to the classical projection methods implemented in R. Yet, the k-NN method also achieves a coverage of more than 90% (and close to the nominal 95% that it targets), and the prediction interval that it provides is thus more reliable than that of other methods.

**Under-ascertainment bias.** Having predicted the daily incidence rate in the weeks leading to the events, to correctly estimate the number of ticket holders that are likely to be contaminated, it is important to correct this prevalence estimate for any under-reporting bias. The under-ascertainment bias refers to the fact that the reported COVID cases are in fact an under-estimation of the actual number of cases, due to either


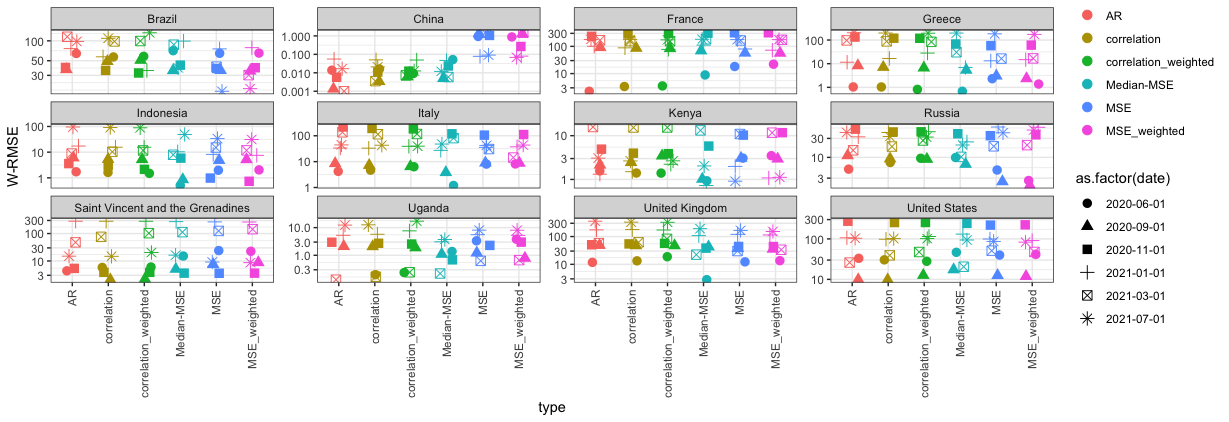


Figure 7: Performance of the different methods for predicting the epidemics trajectories (subset of countries around the world). The MSE performs the best on average and according to 95^th^ percentile, but there are differences in performance across time and countries.


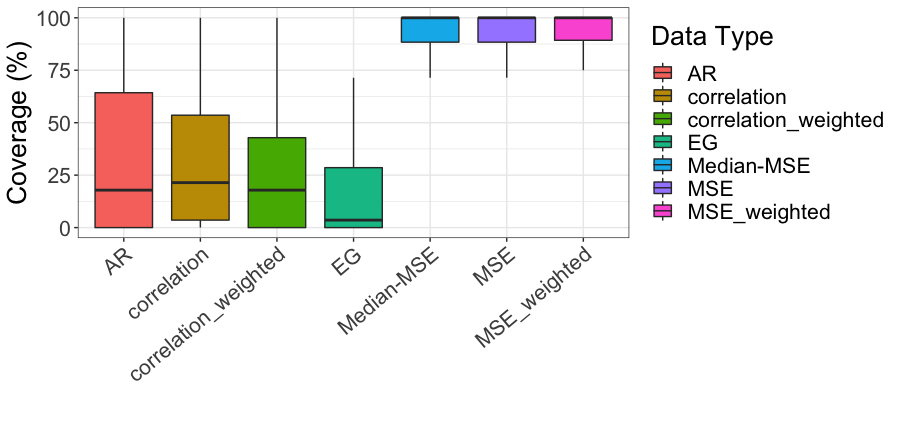
Figure 8: Performance of the different methods for predicting the epidemics trajectories (subset of countries around the world) for coverage

asymptomatic cases or limited testing capacities. In order to compute the appropriate correction, we use age-stratified estimates of the Infection-Fatality rate[[1]](#footnote-1). The method provided at this link has the advantage of computing robust IFR estimates by leveraging data from countries around the world, and adjusting for their demographic makeup. The actual number of cases is then computed as:

$$\text{bias}\left[ t-\text{lag} \right]=\frac{cases\left[ t-lag \right]}{\text{deaths}\left[ t \right]}\times\text{IFR}$$

Indeed, since deaths are offset compared to the incidence rate, reported new cases must be compared to deaths roughly three weeks later (according to CDC reports). The under-ascertainment in the case of Britain is plotted in Figure 6a.

**Step b. Estimating the Number of People who will escape the screening protocol.** For the sake of clarity and to make this appendix self-contained, we repeat here the discussion of the screening protocol provided in the main text, but provide additional detail on the estimation procedure. For an infectious individual to attend the event in spite of the CAPACITY study’s screening protocol, they must (a) have no COVID-like symptoms or fail to report them on the morning of the event, (b) receive a (false) negative result during antigen testing D = 2 days prior to the event, and (c) be contagious (rather than simply infected) at the time of the event.

*(i)* *Symptoms-Check Failure.* Indeed, one of the main challenges associated with the COVID-19 crisis is the number of asymptomatic cases - that is, infected individuals that do not express symptoms and are thus unaware of their potential infectiousness. This group encompasses people that are either pre-symptomatic or completely asymptomatic during the course of their illness – the latter are estimated to represent roughly 25% of all cases [17]. To account for this temporal dependency, we use estimates of the incubation period (defined as the number of days between infection and symptom onset) from McAloon et al. [17] and data on symptoms duration from van Kampen et al. [18] to estimate the probability for a ticket holder infected *s* days before the event to exhibit symptoms on the day of the event. We rely on simulations to estimate this probability distribution, finding estimates of the time to symptom onset by randomly generating an incubation period using data from McAloon et al. [17], and sampling a symptom duration from van Kampen et al. [18]. The resulting density plot is displayed in red in Figure 3a.

*(ii) Antigen test failure.* The sensitivity of COVID tests depends heavily on the time since infection, and whether these are the gold-standard PCR or Lateral Flow Antigen Assays [18,19]. Moreover, studies have shown that LFA tests have much lower sensitivity on asymptomatic individuals than symptomatic: in particular, according to a recent CDC report [20], Rapid Antigen testing has 80% sensitivity on symptomatic individuals, but only 40% sensitivity on asymptomatic individuals. Coupling the sensitivity estimates [20, 21] with the distribution of incubation period and estimated percentage of asymptomatic cases [17, 18], for each individual infected at day *k* taking an antigen test *D* days before the event, the probability of getting through the filtering protocol is thus given by the formula:

$$\mathbb{P}\left[ \text{Protocol fails} | \text{Contaminated at time }k \right]=\left( 1-s_{t-k-D}^{\text{(symptomatic)}} \right)\left( p_{SC}\times p_{t-k}^{\left( \text{symptom} \right)}+\left( 1-p_{t-k}^{\left( \text{symptom} \right)} \right) \right)+p^{\left( \text{asymptomatic} \right)}\times\left( 1-s^{\left( \text{asymptomatic} \right)} \right)$$

where $s_{t-k-D}^{\text{(sym}\text{ptomatic}\text{)}}$ and $s^{\left( \text{asymptomatic} \right)}$ are respectively the sensitivities of the test taken *D* days before the event for a symptomatic participant infected $t-k$ days before the event and an asymptomatic individual. The parameter $p_{t-k}^{\left( \text{symptom} \right)}$ denotes the probability for a symptomatic individual to exhibit symptoms *t k* days after infection, whereas $p^{\left( \text{asymptomatic} \right)}$ is the probability of being asymptomatic. Finally, the variable $p_{SC}$ denotes the probability of the Symptoms Check failing — namely, that the participant does not want to report their symptom. Currently, this probability is set by default in our model to 50%, and we provide in our interactive dashboard the option to choose other levels. As the CAPACITY study gathers more behavioural data on the participants, we hope to improve this estimate. However, we study the sensitivity of our analysis to the choice of this variable in Appendix C, and show that in view of the total uncertainty surrounding other parameters in the model, the choice of this parameter does not severely affect the robustness of the results.

Figure 3b in the main text shows the probability of the failure of the screening protocol as a function of days after infection. This curve was also simulated by sampling: we model the uncertainty in the sensitivity through a set of Monte Carlo simulations, in which, for each simulation: (a) we sample a random sensitivity from a beta distribution, with parameters chosen to match the uncertainty intervals provided in [19], and (b) associate these random sensitivity to the probability of having symptoms and failing to report them. The shaded areas in Figure 3b denote the uncertainty around this estimate due to the variability of the incubation time.

1. *Estimating Infectiousness.* Infectiousness is a function of time since infection. Many articles in the literature have in particular estimated infectiousness to be at its peak within the first five days after symptom onset. However, very few reports provide an in-depth description of infectiousness as a function of time since infection. To this end, in this paper, we combine data from multiple sources. In particular, we rely on the data from Singanayagam et al [21]. Indeed, in this article, the authors study infectiousness as a function of time since symptom onset which they estimate by looking at the percentage of viable cultures that they can obtain from samples collected at various intervals before and after symptom onset. Since our goal is to consider infectiousness as a function of time since infection (rather than symptom onset), we combine this data with the estimated distribution of incubation length (duration between the date of infection, and date of symptom onset). One of the main issues in converting the data from [21] lies in the long tails of this distribution, which extrapolates from the data and allows samples to be highly contagious up to 10 days before infection, Since Singanayagam et al [21] have very few cases past 4 days prior since infection, we threshold infectiousness to 0 to be consistent with the estimates by He et al [22]. We compound the distribution of incubation and infectiousness by a probability of the incubation length, yielding a probability of infectiousness *s* days after infection such that:

$$\text{Infectiousness}_{s}=\sum_{i=1}^{\text{max}(15,s-1)} \mathbb{P}\left[ \text{Incubation period = }i\text{ days} \right]\mathbb{P[}\text{Live culture at day }s-i]$$

2. **Step c: Estimating the number of people at risk.** Finally, the last quantity that we need to impute is the number of people at risk during the event. As described in the main text, this requires a knowledge of the participants’ COVID immunization status, i.e., has the participant already had COVID in the previous year and/or has the participant been vaccinated. This immunization status could be imputed through the combination of information regarding vaccination status as well as additional questions (previous positive test for COVID, symptoms, etc., combined in a model such as in [23]). However, for the sake of simplicity, we only consider here the vaccination status of the participants - thus leaving out the proportion of the population that has had COVID but has not been vaccinated yet. This induces a risk estimate that is biased upward — that is, we do not account for the immunity naturally gained by ticket holders through COVID infection — and as such, is more conservative. When imputing the risk for Horizon 1 (a few weeks before the event, and without any ticket holders’ information), we impute the event’s crowd immunity level using linear regression. In other words, we assume that the number of new vaccinations (first and second dose) grows linearly each day, which amounts to assuming that vaccinations are operating at capacity. Figure 7 shows a plot of the cumulative number of first and second doses in the UK as a function of time, highlighting a good fit between the linear model and the actual observations. We note though that as vaccination levels are increasing, the linear model will potentially have to be modified: after a certain proportion of the population has been vaccinated, vaccinations could stop operating at capacity since the remainder of the population could either have difficulties in gaining access to the vaccine, or could be opposed to the vaccine altogether. However, at the time of writing, the linear fit seems to be a good fit. Having imputed the rate of new vaccinations $\pi_{s=1\ldots t}$ in the days leading to the event, we turn to the estimation of the number of individuals that are likely to be susceptible. Recent reports indicate that vaccine-acquired immunity is a function of both time since vaccination and number of doses [24]. To compute the effective number of participants at risk in the event, we use a compound Poisson distribution: on each day *s* in the weeks leading to the event, the number *X* of new participants vaccinated (having either their first or second dose) is expressed as a Poisson($\pi_{s}^{\left( \text{dose} j \right)}$), where $j\in1,2$. Each of these newly vaccinated individuals then has a probability $\rho_{t-s}^{\left( \text{dose} j \right)}$ of being immune, depending on the date and dose *j* that they have received. The resulting number of immune people Z attending the event thus follows a Poisson model with rate $Z\simℲr\{Poisson\}(\sum_{j=1}^{2} \sum_{s=1}^{T} \pi_{s}^{\left( \text{dose} j \right)}\rho_{t-s}^{\left( \text{dose} j \right)}$.

However, as the number of vaccination increases, we expect the probability of the participants being immune to increase. In this case, we simply replace the Poisson binomial with a binomial, for various values of $\rho_{t-s}^{\left( \text{dose} j \right)}$ to allow the uncertainty around immunity to percolate through the model.

(A)

(B)

(A)


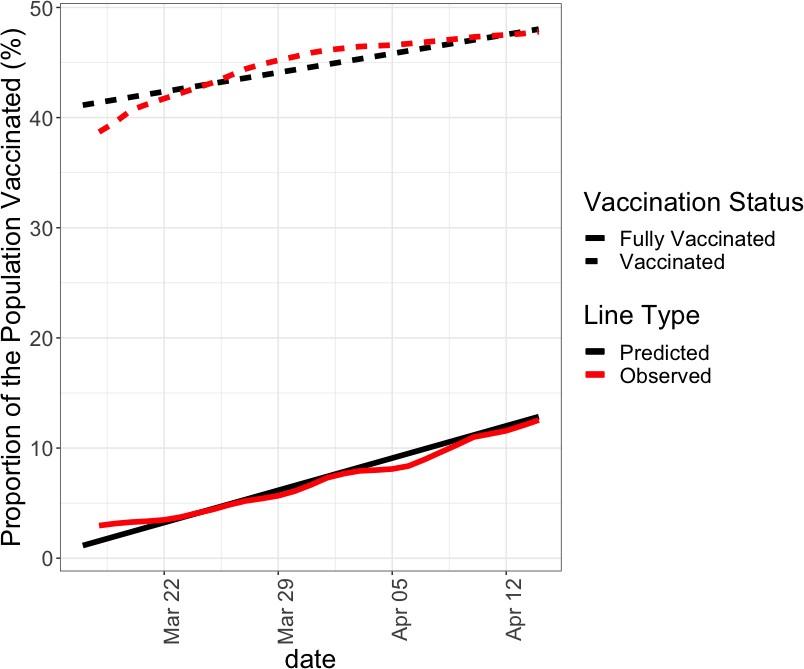

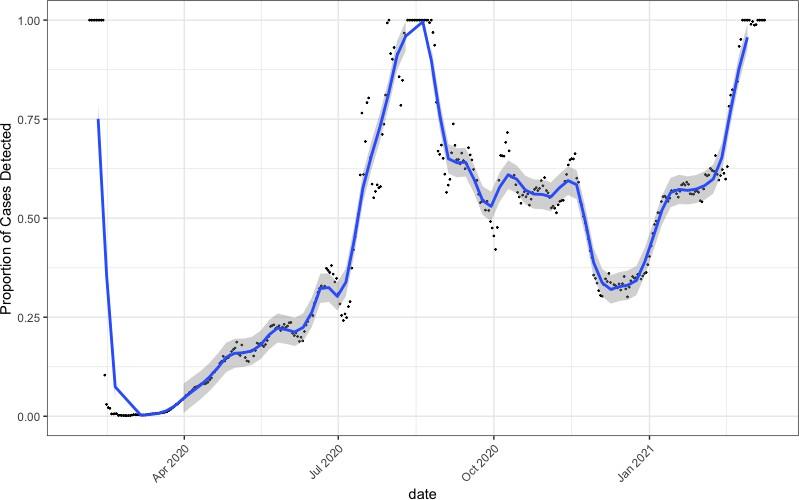


(a) Ascertainment rate for the United Kingdom. The y-axis denotes the ratio of detected cases to cases predicted by multiplying a 3-week shifted death rate by a calculated infection-fatality rate for the U.K. When the values are less than one it suggests that many of the actual cases are not being detected, and when the values are greater than one it suggests that either a substantial proportion of cases are false positives or that survival from COVID infection has increased from baseline predictions, due to better treatments or a greater bias towards infection of individuals at lower risk of death.
(b) Vaccination rate: comparison of the actual rates, and the ones predicted by a linear model for the United Kingdom. The vaccination rates (both first and second dose) seem to be well approximated by a linear regression model (using than time as a covariate), with an associated R^2^ of 0.92. As time progresses, and vaccination rates increase, this linear fit might start becoming less accurate, as problems of vaccination hesitancy or difficulty to access might induce vaccine centers to operate under capacity.

Figure 9: Under-ascertainment rate (left) and vaccination rate (right) in the United Kingdom.

References:

1. Grant A. Dynamics of COVID-19 epidemics: Seir models underestimate peak infection rates and overestimate epidemic duration. medRxiv. Preprint posted April 12, 2020. doi: 10.1101/2020.04.02.20050674.
2. He S, Peng Y, Sun K. Seir modeling of the COVID-19 and its dynamics. Nonlinear Dynamics. 2020; 101:1667-1680. doi: 10.1007/s11071-020-05743-y.
3. Pandey G, Chaudhary P, Gupta, R, Pal S. Seir and regression model based COVID-19 outbreak predictions in india. medRxiv. Preprint posted April 3, 2020. doi: 10.1101/2020.04.01.20049825.
4. Wu JT, Leung K, Leung GM. Nowcasting and forecasting the potential domestic and international spread of the 2019-ncov outbreak originating in Wuhan, China: a modelling study. The Lancet. 2020; 395(10225). doi: [10.1016/S0140-6736(20)30260-9](http://dx.doi.org/10.1016/S0140-6736(20)30260-9).
5. Zhao S, Chen H. Modeling the epidemic dynamics and control of COVID-19 outbreak in China. Quantitative Biology. 2020; 11:1-9. doi: [10.1007/s40484-020-0199-0](https://dx.doi.org/10.1007%2Fs40484-020-0199-0).
6. Akbarpour M, Cook C, Marzuoli A, et al. Socioeconomic network heterogeneity and pandemic policy response. NBER Working Paper. Preprint posted June 2020. doi: 10.3386/w27374.
7. Chang SL, Harding N, Zachreson C, Cliff OM, Prokopenko M. Modelling transmission and control of the COVID-19 pandemic in Australia. Nature. 2020; 11(5710). doi: 10.1038/s41467-020-19393-6.
8. Kai D, Goldstein G, Morgunov A, Nangalia V, Rotkirch A. Universal masking is urgent in the COVID-19 pandemic: Seir and agent based models, empirical validation, policy recommendations. arXiv. Preprint posted online April 22, 2020. doi: [10.13140/RG.2.2.21662.08001](http://dx.doi.org/10.13140/RG.2.2.21662.08001)
9. Rockett RJ, Arnott A, Lam C, et al. Revealing COVID-19 transmission in Australia by SARS-CoV-2 genome sequencing and agent-based modeling. Nature Medicine. 2020; 26(9):1398-1404. doi: 10.1038/s41591-020-1000-7.
10. Silva PCL, Batista PVC, Lima HS, Alves MA, Guimarães FG, and Silva RCP. COVID-abs: An agent-based model of COVID-19 epidemic to simulate health and economic effects of social distancing interventions. Chaos, Solitons & Fractals. 2020; 139:110088. doi: [10.1016/j.chaos.2020.110088](https://dx.doi.org/10.1016%2Fj.chaos.2020.110088).
11. Wallinga J, Lipsitch M. How generation intervals shape the relationship between growth rates and reproductive numbers. Proceedings of the Royal Society B: Biological Sciences. 2007; 274(1609):599-604. doi: 10.1098/rspb.2006.3754.
12. Dietz K. The estimation of the basic reproduction number for infectious diseases. Statistical Methods in Medical Research. 1993; 2(1):23-41. doi: 10.1177/096228029300200103
13. He J, Guo Y, Mao, R, Zhang J. Proportion of asymptomatic coronavirus disease 2019: A systematic review and meta-analysis. Journal of medical virology. 2021; 93(2):820-830. doi: 10.1002/jmv.26326.
14. Obadia T, Haneef, R, Boëlle P. The R0 package: a toolbox to estimate reproduction numbers for epidemic outbreaks. BMC Medical Informatics and Decision Making. 2012; 12(1):1-9. doi: 10.1186/1472-6947-12-147.
15. Kamvar ZN, Cai J, Pulliam JRC, Schumacher, J, Jombart T. Epidemic curves made easy using the r package incidence. F1000Research. 2019; 8:139. doi: [10.12688/f1000research.18002.1](https://dx.doi.org/10.12688%2Ff1000research.18002.1).
16. R Epidemics Consortium. Projections. Available from <https://www.repidemicsconsortium.org/projections/index.html>.
17. McAloon C, Collins A, Hunt K, et al. Incubation period of COVID-19: a rapid systematic review and meta-analysis of observational research. BMJ Open. 2020; 10(8):e039652. doi: 10.1136/bmjopen-2020-039652.
18. van Kampen JJA, van de Vijver DAMC, Fraaij PLA, et al. Duration and key determinants of infectious virus shedding in hospitalized patients with coronavirus disease-2019 (COVID-19). Nature Communications. 2021; 12:267. doi: [10.1038/s41467-020-20568-4](https://doi.org/10.1038/s41467-020-20568-4).
19. Kucirka LM, Lauer SA, Laeyendecker O, Boon, D, Lessler J. Variation in false-negative rate of reverse transcriptase polymerase chain reaction-based SARS-CoV-2 tests by time since exposure. Annals of Internal Medicine. 2020 173(4):262-267. doi: [10.7326/M20-1495](https://dx.doi.org/10.7326%2FM20-1495).
20. Pray IW. Performance of an antigen-based test for asymptomatic and symptomatic SARS-CoV-2 testing at two university campuses—Wisconsin, September-October 2020. Morbidity and Mortality Weekly Report. 2021; 69(5152):1642-1647. doi: 10.15585/mmwr.mm695152a3.
21. Singanayagam A, Patel M, Charlett A, et al. Duration of infectiousness and correlation with RT-PCR cycle threshold values in cases of COVID-19, England, January to May 2020. Eurosurveillance. 2020; 25(32):2001483. doi: [10.2807/1560-7917.ES.2020.25.32.2001483](https://doi.org/10.2807/1560-7917.ES.2020.25.32.2001483).
22. He X, Lau EHY, Wu P, et al. Temporal dynamics in viral shedding and transmissibility of COVID-19. Nature Medicine. 2020; 26(5):672-675. doi: 10.1038/s41591-020-0869-5.
23. Donnat C, Miolane N, Bunbury F, Kreindler J. A Bayesian hierarchical network for combining heterogeneous data sources in medical diagnoses. Machine Learning for Health. 2020; 136:53-84.
24. Public Health England. Impact of COVID-19 vaccines on mortality in England: December 2020 to March 2021. 2021. https://assets.publishing.service.gov.uk/government/uploads/system/uploads/attachment_data/file/977249/PHE_COVID-19_vaccine_impact_on_mortality_March.pdf/.

1. The IFR have been taken from the following data source: https://github.com/mbevand/covid19-age-stratified-ifr [↑](#footnote-ref-1)
