# Supplementary material for: Predicting COVID-19 Transmission to Inform the Management of Mass Events: Model-Based Approach
Source: JMIR Public Health Surveill. 2021 Dec 1;7(12):e30648. doi: 10.2196/30648 (PMC8638785; doi:10.2196/30648)
Supplement: Multimedia Appendix 2 [file publichealth_v7i12e30648_app2.docx]

# Transmission

# In this appendix, we describe in greater details the model and assumptions made by the Jimenez aerosol transmission model proposed in [1, 2] and used to model transmission dynamics in the main text.

**The issue of COVID transmission.** As discussed in the main text, the precise mechanisms by which COVID- 19 is transmitted are still unclear. Aside from direct physical contact, experts continue to debate the significance of the following two main routes of infection:

1. *Droplet transmission.* In this scenario, transmission happens through the inhalation of droplets (particles of 5 to 10 *µ*m in diameter [3]), and typically occurs when a person is in close proximity (within 1 meter) with someone who has respiratory symptoms (e.g., coughing or sneezing). In the context of live events, modelling this specific transmission route involves (a) modelling the distribution of the number of close contacts between infectious and susceptible ticket holders during the event and (b) modelling the transmission probability foreach close contact. The latter is a function of the proximity between participants and the amount of time spent in the vicinity of the infectious individual.
2. *Airborne transmission.* Increasing concerns around airborne transmission have been raised by a number of experts over the past few months [4, 5]. Airborne transmission refers to the presence of the virus within droplet nuclei remaining in the air for long periods of time and with the potential to travel long distances [4] and penetrate more deeply in respiratory tracts. Airborne transmission has been estimated to be nearly 19 times more likely indoors than outdoors [6]. In the context of large public events, this transmission route thus has more diffusive power and hence could explain several super-spreader events (SSEs) [7] making it a major cause for concern [2,4,8-15]. In this setting, infection risk is typically modelled using one of two distinct models: Wells-Riley equations and the dose-response model [16,17]. First introduced by Riley in 1978 in a study of measles outbreak [18], the Wells-Riley equations are based on the concept of a hypothetical infectious unit called a "quantum of infection" [18,19], defined as the number of infectious airborne particles required to infect a person. Quanta aim to capture in a single parameter the rate of emission of viral particles in exhaled breath, the infectivity of the viruses upon emission, the particle size distribution of the emissions, the deposition efficiency and deposition location in the respiratory tract of the susceptible person, as well as the probability that deposition leads to infection. The dose-response model aims to describe more directly the effect on organisms from the exposure to different doses of chemicals, drugs, radiation, biological agents, or other stressors — and more recently to assess the infection risk of airborne transmissible pathogens. The review by Sze To et al [16] provides an in-depth comparison of the two models. In the context of COVID transmission, one of the main limitations of the dose-response model is that it relies on infectious dose data to derive the dose-response relationship. By contrast, many of the parameters in the Wells-Riley equation can be approximated and has thus been favored by many experts in the field [2,20,23].

**Choice of Transmission Route for this Model.** While droplet emission is undeniably a source of concern and a major source of transmission, simple safety precautions such as mask wearing have been shown to efficiently control this transmission source [1, 21]: it is estimated that face masks can block 80% of exhaled droplets and reduce inhaled droplets by up to 50%, and so on average reduce the transmission probability by 70%. Conversely, the evidence concerning the efficiency of standard protective equipment in filtering aerosol droplets varies widely across studies probably due to “variation in experimental design and particle sizes analyzed” [22]. Airborne transmission in indoor settings can thus represent one of the main risk factors in live events, which we focus on modelling using the aerosol model proposed by Jimenez [1, 2]. The Jimenez aerosol transmission model [1, 2] is indeed currently one of the only COVID-transmission models that provides enough granularity to quantify the risk associated with an event. This recognized model has been used several times in the literature over the course of the pandemic, including to allow in-class teaching at the University of Illinois at Chicago [11]. Based on the Wells-Riley model [18,19,16], this estimator calibrates the quanta to known transmission events, and takes into account important factors to compute a risk estimate, including event-specific (number of people, local prevalence, etc.) and venue-specific variables (ventilation rate, size of the venue, UV exposure).

A core principle behind the Wells-Riley model is the notion of “quantum of infection”. Exposure to one quantum of infection gives an average probability of $63\%=1-e^{-1}$ of becoming infected (essentially an infectious dose 63%, ID63) [23]. The crux of the Wells-Riley equation consists of its modelling of the probability of infection *P_I_* as a function of the ventilation, inhalation rates and quanta generation rates:

$P_{I}=1-e^{-\frac{Iqpt}{Q}} (*)$

where $n_{I}$ is the number of infectors, $p$ is the pulmonary ventilation rate of a person, $q$ is the quanta generation rate, $t$ is the exposure time interval, and $Q$ is the room ventilation rate with clean air. Note that this equation is not dimensionless. As explained by Rudnick and Milton [23], $q$ represents the generation rate of infectious doses, not organisms or infectious particles; it is the average infectious source strength of infected individual. Thus, the exponential form of the probability equation reflects the probability of a susceptible person in the room inhaling at least one quanta, based on a Poisson distribution of the number of discrete quanta inhaled by a susceptible person present in the space, given a certain aerosol quanta concentration in the room and an inhalation time.

One of the advantages of the Wells-Riley model is that many extensions have been studied, allowing the incorporation of additional influencing factors. In particular, the effect of respiratory protection can be considered by multiplying the term in the exponential by a fraction [24-26]:

$$P_{I}=1-\text{exp}\left\{ -R\frac{Iqpt}{Q} \right\} \left( ** \right)$$

where $R$ is a number between 0 and 1 representing the fraction of particle penetration of the respiratory protection (it is in particular equal to 1 when no respirator is used). Other variables, such as the ultra-violet irradiation, particle filtration have been taken into account in the Wells-Riley equation through the equation:

$$P_{I}=1-\text{exp}\left\{ -\frac{Iqpt}{Q+\lambda_{UV}V+Q_{r}\eta_{r}} \right\} (***)$$

where $\lambda_{UV}$ is rate coefficient of inactivation by ultraviolet irradiation, $V$ is the room volume, $Q_{r}$ is the flow rate to the filter, and $\eta_{r}$is the filtration efficiency.

As suggested by Jimenez et al [1,2], instead of modelling each of parameters in the Wells Riley equation explicitly, we can use the concept of quanta, and calibrate the emission rate to known outbreaks of the disease. We base the following description of the model as detailed by Jimenez et al [1,2]. This model relies on the computation of three main components:

1. **The Quanta Emission Rate.** The quanta emission rate can be interpreted as the number of quanta emitted by unit of time by a single infectious participant. It can be modelled as:

$$q_{e}= \text{Quanta Exhalation Rate} \times(1 - \text{Mask Efficiency}\mathbb{*P}\left[ \text{Wear Mask} \right]) \times n_{\text{infectious}}$$

2. **The Quanta Concentration Rate.** The quanta concentration rate *q_c_* is computed as:

$$q_{c}=\frac{q_{e}}{\text{Loss}\times\text{Volume}}\times\left( 1 - \frac{1}{\text{Loss}\times\text{Duration}}\times\left( 1-\text{exp}\left\{ -\text{Loss}\times\text{Duration} \right\} \right) \right)$$

where $q_{e}$ is the quanta emission rate and $n_{\text{infectious}}$ is the number of infectious people at the event. The loss corresponds to the first order loss:

The term "Decay" corresponds here to the decay rate of the virus, and is a function of the UV, temperature, and relative humidity of the event. The Decay rate per hour is computed according to the following formula [27,28] for which an open-source calculator is available online[[1]](#footnote-1):

$$\text{Decay}=\left( 7.57+1.41\times\frac{T-20.54}{10.66}+0.022\times\frac{RH-45.24}{28.67}+7.55\times\frac{0.185\times UV-50}{50}+\frac{T-20.54}{10.66}\times\frac{0.185\times UV-50}{50}\times1.40 \right)\times60$$

where T is the temperature and UV is the UV index. The tool is valid for the following ranges of conditions: 10 to 30°C (50-86°F), 20-70% relative humidity, and UV indices of 0-10. For live events in an indoor setting, the UV parameter should be set to 0.

We have checked that our computations are aligned with the figures provided in the references.

1. **The Quanta Inhalation Rate.** The quanta inhalation rate is computed as:

Values for the Mask Inhalation Efficiency, as well as for the breathing rate (which varies by activity) can be found at the bottom of this page. We have used the tables suggested by Jimenez [1], and refer to the sources he suggests for a more fine-grain estimate of what these should be[[2]](#footnote-2).


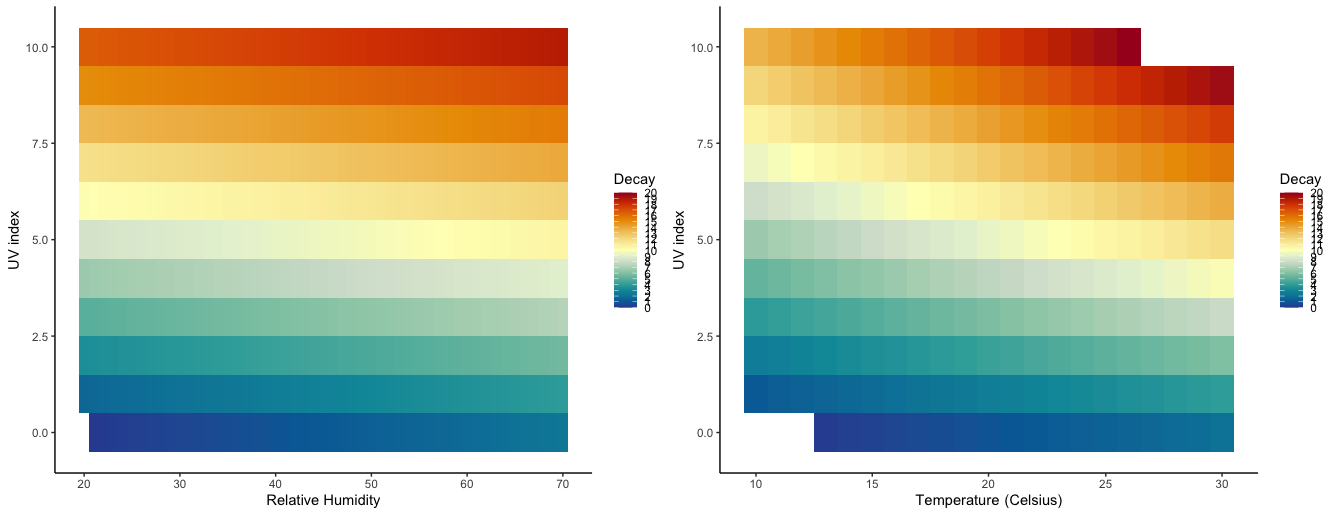


**(B)**

**(A)**

Figure 10: Decay rate of the virus, reproduced from [85, 86], and as a function of humidity, UV index, as well as temperature. NA values appear as transparent tiles. **Panel A:** Decay rate as a function of UV and relative humidity, for a temperature of 20° Celsius. **Panel B:** Decay rate as a function of UV and temperature (in Celsius, for a relative humidity of 40%).

**Ventilation Rates.** The ventilation rate per person is computed as:

$$\text{Ventilation}=\frac{1}{n_{\text{tot}}}\times\text{Volume}\times\text{Ventilation with outside air}\times\frac{1000}{3600}$$

where $n_{\text{tot}}$ is the total number of participants, and the ventilation rate is measured in $L^{-1}/s$ and depends on the activity. Again, for the sake of completeness, we have included in Appendix E (Figure 15) the table from the AHRAE (American Society of Heating, Refrigerating and Air-Conditioning Engineers) listing all the activities as well as their corresponding ventilation rates[[3]](#footnote-3) that were suggested by Jimenez [1] to perform the computations.

**References:**

1. Jimenez. Aerosol transmission model, 2020. Available at <https://docs.google.com/spreadsheets/d/16K1OQkLD4BjgBdO8ePj6ytf-RpPMlJ6aXFg3PrIQBbQ/edit#gid=519189277>, Accessed August 3^rd^ 2021.
2. Miller SL, Nazaroff WW, Jimenez JL, et al. Transmission of SARS-CoV-2 by inhalation of respiratory aerosol in the Skagit Valley Chorale superspreading event. Indoor air. 2021; 31(2):314-323. doi: 10.1111/ina.12751.
3. World Health Organization. Modes of transmission of virus causing COVID-19: implications for ipc precaution recommendations. 2020. https://www.who.int/news-room/commentaries/detail/modes-of-transmission-of-virus-causing-covid-19-implications-for-ipc-precaution-recommendations/.
4. Morawska L, Milton DK. It is time to address airborne transmission of coronavirus disease 2019 (COVID-19). Clinical Infectious Diseases. 2020; 71(9):2311-2313. doi: [10.1093/cid/ciaa939](https://doi.org/10.1093/cid/ciaa939).
5. Lewis D. Is the coronavirus airborne? experts can’t agree. Nature. 2020; 580(7802):175. doi: 10.1038/d41586-020-00974-w.
6. Nishiura H, Oshitani H, Kobayashi T, et al. Closed environments facilitate secondary transmission of coronavirus disease 2019 (COVID-19). medRxiv. Preprint posted April 16, 2020. doi: 10.1101/2020.02.28.20029272.
7. Hamner L. High SARS-CoV-2 attack rate following exposure at a choir practice—Skagit county, Washington, March 2020. Morbidity and Mortality Weekly Report. 2020; 69(19):606-610. doi: 10.15585/mmwr.mm6919e6.
8. Asadi S, Bouvier N, Wexler, AS, Ristenpart WD. The coronavirus pandemic and aerosols: Does COVID-19 transmit via expiratory particles? Aerosol Science and Technology. 2020; 54(6):635-638. doi: [10.1080/02786826.2020.1749229](https://doi.org/10.1080/02786826.2020.1749229).
9. Zhang R, Li Y, Zhang AL, Wang, Y, Molina MJ. Identifying airborne transmission as the dominant route for the spread of COVID-19. Proceedings of the National Academy of Sciences. 2020; 117(26):14857-14863. doi: 1[0.1073/pnas.2009637117](https://doi.org/10.1073/pnas.2009637117).
10. Bhagat RK, Wykes MSD, Dalziel SB, and Linden PF. Effects of ventilation on the indoor spread of COVID-19. Journal of Fluid Mechanics. 2020; 903:F1. doi:10.1017/jfm.2020.720.
11. Elbanna A, Wong GN, Weiner ZJ, et al. Entry screening and multi-layer mitigation of COVID-19 cases for a safe university reopening. medRxiv. Preprint posted September 2, 2020. doi: [10.1101/2020.08.29.20184473](https://doi.org/10.1101/2020.08.29.20184473).
12. Buonanno G, Stabile L, Morawska L. Estimation of airborne viral emission: Quanta emission rate of SARS-CoV-2 for infection risk assessment. Environment International. 2020; 141:105794. doi: [10.1016/j.envint.2020.105794](https://doi.org/10.1016/j.envint.2020.105794).
13. Fennelly KP. Particle sizes of infectious aerosols: implications for infection control. The Lancet Respiratory Medicine. 2020; 8(9):914-924. doi: [10.1016/S2213-2600(20)30323-4](https://doi.org/10.1016/S2213-2600(20)30323-4).
14. Majra D, Benson J, Pitts J, Stebbing J. Sars-cov-2 (COVID-19) superspreader events. Journal of Infection. 2021; 82(1):36-40. doi: [10.1016/j.jinf.2020.11.021](https://dx.doi.org/10.1016%2Fj.jinf.2020.11.021)
15. He X, Lau EHY, Wu P, et al. Temporal dynamics in viral shedding and transmissibility of COVID-19. Nature Medicine. 2020; 26(5):672-675. doi: 10.1038/s41591-020-0869-5.
16. To GNS, Chao CYH. Review and comparison between the wells-riley and dose-response approaches to risk assessment of infectious respiratory diseases. Indoor Air. 2010; 20(1):2-16. doi: 10.1111/j.1600-0668.2009.00621.x.
17. Noakes CJ, Beggs CB, Sleigh PA, and Kerr KG. Modelling the transmission of airborne infections in enclosed spaces. Epidemiology and Infection. 2006; 134(5):1082-1091. doi: [10.1017/S0950268806005875](https://dx.doi.org/10.1017%2FS0950268806005875).
18. Riley EC, Murphy G, Riley RL. Airborne spread of measles in a suburban elementary school. American Journal of Epidemiology. 1978; 107(5):421-432. doi: 10.1093/oxfordjournals.aje.a112560.
19. Wells WF. Airborne contagion and air hygiene. An ecological study of droplet infections. Cambridge, MA: Harvard University Press; 1955.
20. Harrichandra A, Ierardi, AM, Pavilonis B. An estimation of airborne SARS-CoV-2 infection transmission risk in New York City nail salons. Toxicology and Industrial Health. 2020; 36(9):634-643. doi: [10.1177/0748233720964650](https://doi.org/10.1177%2F0748233720964650).
21. Peeples L. What the data say about wearing face masks. Nature. 2020; 586:186-189. doi: 10.1038/d41586-020-02801-8.
22. Brooks JT, Butler JC. Effectiveness of mask wearing to control community spread of SARS-CoV-2. JAMA. 2021; 325(10):998-999. doi: 10.1001/jama.2021.1505.
23. Rudnick SN, Milton DK. Risk of indoor airborne infection transmission estimated from carbon dioxide concentration. Indoor Air. 2003; 13(3):237-245. doi: 10.1034/j.1600-0668.2003.00189.x.
24. Fennelly KP, Nardell EA. The relative efficacy of respirators and room ventilation in preventing occupational tuberculosis. Infection Control and Hospital Epidemiology. 1998; 19(10):754-759. doi: 10.1086/647719.
25. Nazaroff WW, Nicas M, Miller SL. Framework for evaluating measures to control nosocomial tuberculosis transmission. Indoor Air. 1998; 8(4):205-218. doi: [10.1111/j.1600-0668.1998.00002.x](https://doi.org/10.1111/j.1600-0668.1998.00002.x).
26. Nicas M. An analytical framework for relating dose, risk, and incidence: an application to occupational tuberculosis infection. Risk Analysis. 1996; 16(4):527-538. doi: 10.1111/j.1539-6924.1996.tb01098.x.
27. Schuit M, Ratnesar-Shumate S, Yolitz J, et al. Airborne SARS-CoV-2 is rapidly inactivated by simulated sunlight. The Journal of Infectious Diseases. 2020; 222(4):564-571. doi: [10.1093/infdis/jiaa334](https://doi.org/10.1093/infdis/jiaa334).
28. Dabisch P, Schuit M, Herzog A, et al. The influence of temperature, humidity, and simulated sunlight on the infectivity of SARS-CoV-2 in aerosols. Aerosol Science and Technology. 2021; 55(2):142-153. doi: [10.1080/02786826.2020.1829536](https://doi.org/10.1080/02786826.2020.1829536).

1. This calculator can be found at the following link: [https://www.dhs.gov/science-](http://www.dhs.gov/science-and-technology/)and-[technology/](http://www.dhs.gov/science-and-technology/)sars-airborne-calculator [↑](#footnote-ref-1)
2. Link to the EPA website: https://www.epa.gov/expobox/exposure-factors-handbook-chapter-6 [↑](#footnote-ref-2)
3. Link to the ASHRAE tables: https://ashrae.iwrapper.com/ASHRAE_PREVIEW_ONLY_STANDARDS/STD_621_2019 [↑](#footnote-ref-3)
