# Supplementary material for: Predicting COVID-19 Transmission to Inform the Management of Mass Events: Model-Based Approach
Source: JMIR Public Health Surveill. 2021 Dec 1;7(12):e30648. doi: 10.2196/30648 (PMC8638785; doi:10.2196/30648)
Supplement: Multimedia Appendix 3 [file publichealth_v7i12e30648_app3.docx]

# Sensitivity Analysis

Whilst we have based our estimates of the different parameters used in this model on the literature, and tried to incorporate estimates of their uncertainty to try and correctly estimate our confidence in the output of the model, this is nevertheless contingent on several choices on (a) the probability that a ticket holder will lie and fail to report symptoms on the day of the event in order to get into the event, and (b) the efficiency of the masks, and input parameters in the room.

**(a) Probability of lying.** As explained in the introduction, the risk model that we aim to develop has to be context-aware. That is, the estimates of the risk that the model should output should depend (a) on the prevalence at the time of the event and (b) on the ticket holder’s vaccination status. The only input that requires to be determined is the propensity of people to lie if they have symptoms. This is a priori a difficult parameter to estimate, which would be required to be informed by sociology studies. As the CAPACITY study proceeds, this is in particular one of the parameters that we hope to be able to inform better. However, in the absence of information as to what value of that parameter should be set, we propose here a sensitivity analysis to show that the model is in fact relatively weakly sensitive to the choice of this particular parameter. We show the different infectiousness curves corresponding to different values of the parameter *p*_lie_ in Fig. 8 and 9. As shown in Fig. 8, this probability of lying impacts the value of the maximum probability of infectiousness at the event: this value is maximal at 4 days before the event, with a value of 42.1% if the participants never lie, 48% if these participants lie half of the time, and 53% if they always lie. This represents a 25.8% increase in probability from a scenario where participants are considered as completely trustworthy to one where these participants are considered as unreliable. Table 4 quantifies the impact of this parameter in the case of the Royal Albert Hall in order to assess the sensitivity of the entire pipeline to this particular choice of parameter in two situations: low prevalence (August 3rd 2020) and high prevalence (January 18th 2021). As denoted in this table, the impact of this parameter is small in situations where the prevalence is already small. More substantial deviations occur when the prevalence increases, and such deviations are especially important in the tails (here, the median increases by 6 cases (35%) while the 97.5th prediction interval increased by 29% (69 cases) in situations of high prevalence between $p_{\text{lie}}=0$ and $p_{\text{lie}}=1$. While the relative increase (29%) is important, the absolute difference (69 cases) is small compared to the uncertainty in the prevalence (1245 (sd 375) cases).


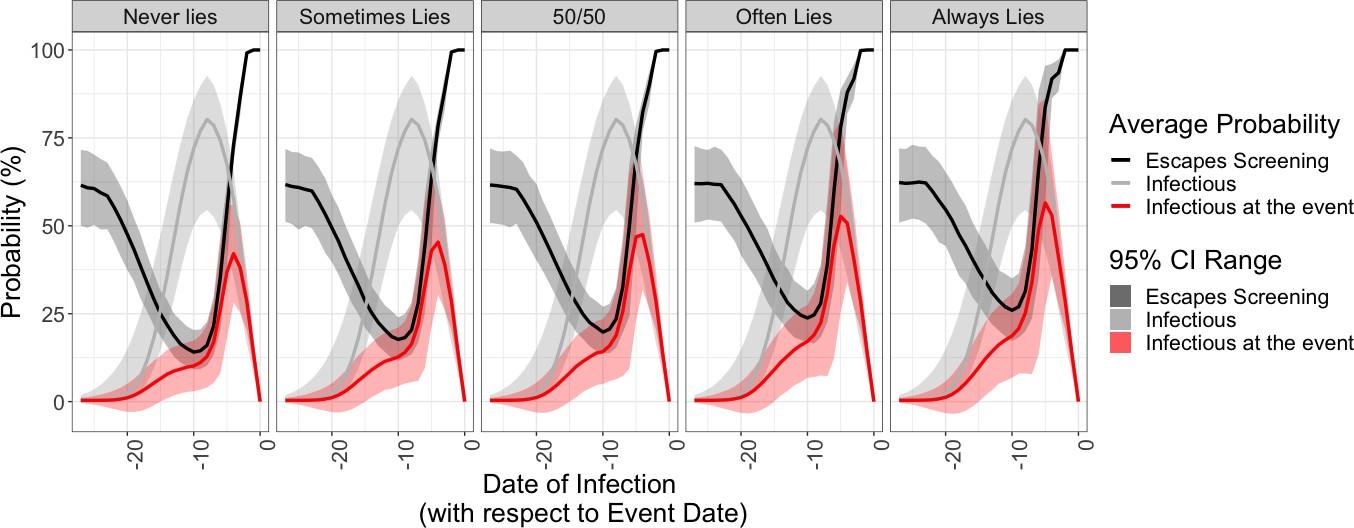


|  | **August 20^th^, 2020** | | | **January 20^th^, 2021** | | |
| --- | --- | --- | --- | --- | --- | --- |
|  | **Never Lies** | **50/50** | **Always Lies** | **Never Lies** | **50/50** | **Always Lies** |
| **Mean** | 0.05 | 0.06 | 0.11 | 1.0 | 2.4 | 2.1 |
| **Median** | 0 | 0 | 0 | 0 | 1 | 1 |
| **97.5th Quantile** | 1 | 1 | 1 | 4 | 10 | 9 |
| **99th Quantile** | 1 | 1 | 2 | 8 | 19 | 16 |

Figure 11: Analysis of the sensitivity of our infectiousness estimation to various values of the probability of lying

_
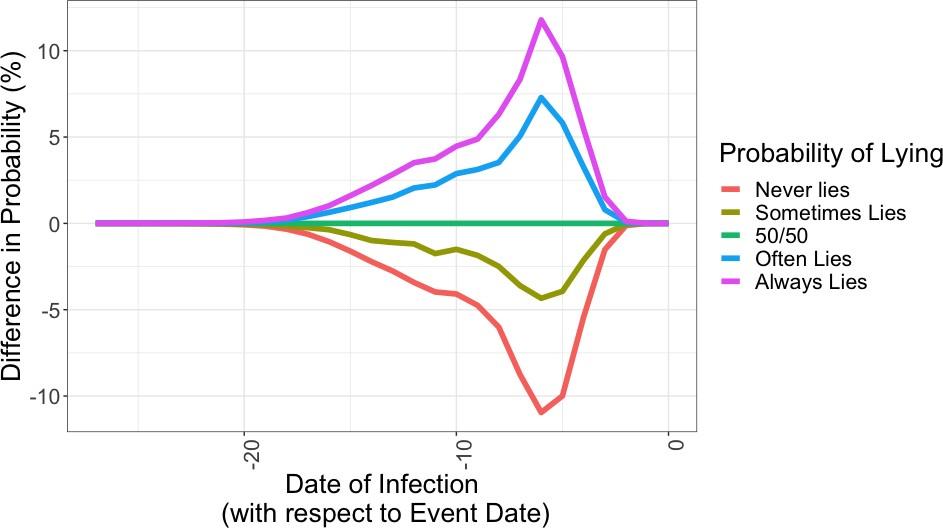
_

Table 4: Quantitative comparison of the sensitivity of the results (number of transmissions at the event) as a function of $p_{SC}$. While the absolute difference between the scenarios remains reasonable for the mean, this difference becomes particularly important in the tails of the distribution (97.5th and 99th quantiles).

Figure 9: Analysis of the sensitivity of our infectiousness estimation to various values of the probability of lying.

**Mask Efficiency.** Another parameter of great importance in the model consists of the effect of the mask efficiency. To this end, we contrast again a scenario with 100% of mandatory mask wearing, but with exhaled efficiency varying from 30, 50, 70, and 90% (the inhaled breathing being fixed efficiency to 50% – the same phenomenon would hold if varying the inhaled breathing efficiency). The results are presented in Table 5. We note the importance sensitivity of the results in the tails of the distribution: the absolute difference (in terms of number of cases) diverges significantly when looking at the 97.5th quantile of the distribution for instance. This highlights the efficiency of masks in limiting superspreading phenomena. We hope to use in particular the results of the CAPACITY study (and behavioural factors such as abidance to mask wearing, preferred type of mask, etc.) as well as the growing literature of mask efficiency to be able to refine our estimate of the mask efficiency. In the meantime, in this paper, we use the conservative 50% efficiency (lower bound provided in [1]).

**Discussion.** In the modelling, we neglect any correlation between ticket holders, however this is unlikely to hold in real life as some might come from the same household. Given participants from the same household would all be rejected if one of them were to test positive, this simplification is likely to be a conservative estimate. Due to the nature of the problem and gaps in what is known about transmission risks, our model does not contend to make precise estimates of the form “we expect x numbers of cases” as a result of the event. Rather, it should be taken as a means of providing a scale of the risk and is best used to make comparisons, for example against a null model in which the event does not occur, but individuals still get infected in the community. As such, our methodology provides a relative quantification of that risk such as “holding the event is expected to increase the number of cases by x% in this pool of participants.” The use of Monte Carlo simulations allows us to account for some uncertainty in our model, and to produce more meaningful risk estimates. As our second goal is to quantify the efficiency of the screening protocol, this pipeline can be run using different screening protocols or testing strategies to determine their efficiency.

|  | **August 20^th^ , 2020** | | | | **January 20^th^ , 2021** | | | |
| --- | --- | --- | --- | --- | --- | --- | --- | --- |
| **Mask Efficiency (%)** | **30** | **50** | **70** | **90** | **30** | **50** | **70** | **90** |
| **Mean** | 0.08 | 0.06 | 0.04 | 0.01 | 3.5 | 2.4 | 1.5 | 0.5 |
| **Median** | 0 | 0 | 0 | 0 | 2 | 1 | 1 | 0 |
| **97.5 Quantile** | 1 | 1 | 0 | 0 | 14 | 10 | 6 | 3 |
| **99th Quantile** | 2 | 1 | 1 | 0 | 27 | 19 | 11 | 5 |

Table 5: Sensitivity of the results to mask efficiency. Note the significant absolute difference in the tails of the distribution.

We also hope to be able to further develop this pipeline through (a) leveraging in-situ data collection and (b) refinement of the models themselves. In particular, using a fully anonymous, post-event questionnaire, we would evaluate participants’ compliance with the screening protocol, as well as with the safety measures at the time of the event. As more events are held, we can quantify the success of our pipeline to provide coverage of the observed number of infections. From the model perspective, we hope to explore further extensions to the current aerosol transmission model, which is based on the Wells-Riley model [2,3] and so assumes uniform mixing of the quanta in the venue. Using information on the potential induced cases (e.g., their relative seating distance), we aim to refine this model by studying ways of accounting for the spatial heterogeneity of the quanta distribution.

**References:**

1. Brooks JT, Butler JC. Effectiveness of mask wearing to control community spread of SARS-CoV-2. JAMA. 2021; 325(10):998-999. doi: 10.1001/jama.2021.1505.
2. Riley EC, Murphy G, Riley RL. Airborne spread of measles in a suburban elementary school. American Journal of Epidemiology. 1978; 107(5):421-432. doi: 10.1093/oxfordjournals.aje.a112560.
3. Wells WF. Airborne contagion and air hygiene. An ecological study of droplet infections. Cambridge, MA: Harvard University Press; 1955.
