# Supplementary material for: Predicting COVID-19 Transmission to Inform the Management of Mass Events: Model-Based Approach
Source: JMIR Public Health Surveill. 2021 Dec 1;7(12):e30648. doi: 10.2196/30648 (PMC8638785; doi:10.2196/30648)
Supplement: Multimedia Appendix 4 [file publichealth_v7i12e30648_app4.docx]

# Risk Communication

**Vaccine passports and widespread antigen tests – a false sense of security?** The use of vaccine passports for international air travel has ignited significant debate in the UK and even more controversial is their use for entry to mass, live events [1]. Notwithstanding the challenges surrounding operational verification of vaccine certification, the ethical implications of excluding those unable or unwilling to be vaccinated from participating in normal social encounters and the resulting implications for social inequities [2], the use of vaccine certification to permit entry to an event will likely significantly overestimate its safety. Vaccinated individuals may still be infected with SARS-CoV-2. Even antigen-test based screenings of ticket holders prior to an event will likely overestimate the safety of the event as some tests will be falsely negative. The definition of what constitutes an admissible level of risk thus poses a difficult conundrum to the live event industry.

**CAPACITY-UK.** The motivating application behind this paper is the CAPACITY study [3]— a partnership between CERTIFIC (a private, remote testing, health status and identify certification service) and Imperial College London – to predict and measure the outcomes of full capacity live events whilst ensuring rigorous abidance by public health and safety measures. Central to this study is the efficiency of pre-event screening by testing all ticket holders using professionally-witnessed rapid at-home antigen tests, and post-event monitoring based on antigen tests, surveys, and safety recommendations. Mass rollout of home-based Lateral Flow Testing to all adults in the UK [4] for twice weekly testing ensures that all households will already have the tests available to them. CAPACITY-UK proposes simply for the tests to be professionally witnessed via the CERTIFIC application to overcome the trust issue, verifying that tests have been collected and conducted to the appropriate standard. In addition to testing (which is susceptible to false positives and negatives), the CAPACITY protocol gathers anonymized information on participant vaccination status, regional address, and a few basic questions regarding individual characteristics (see Fig. 1). The purpose of this additional information is to allow the design of a tailored risk estimation model — both at the participant and at the community level. Such risk estimates are central to the protocol: not only are they necessary in the context of informed consent and communicating to the ticket holders their own level of risk so that they may choose to attend the event, but they are also essential in informing event managers and policy makers on the likelihood of an outbreak. This system potentially allows for the management of full capacity, live events – a crucial parameter for commercial viability of the industry. Moreover, contrary to the issues surrounding vaccination passports, vaccination status would be requested, but not required for attendance – particularly if overall risk of transmission at the event remains within acceptable bounds.

This system potentially allows for the management of full capacity, live events – a crucial parameter for commercial viability of the industry. Moreover, contrary to the issues surrounding vaccination passports, vaccination status would be requested, but not required for attendance – particularly if overall risk of transmission at the event remains within acceptable bounds.

**Timeline Participant flow Model inputs Risk estimate outputs**

Applicants

Overall risk of infections and hospitalisations estimated using case predictions, screening efficacy and transmission dynamics on a hypothetical population

Case predictions, screening efficacy and transmission dynamics on a hypothetical population

Agree to terms of service and consents, purchases ticket, takes survey, books antigen test, loads public health test and trace app

Estimation of the overall risk of infections and hospitalisations

Ticket holder reminded of hygiene, limiting exposure and mask wearing to reduce infection risk

Ticket holder takes virtually-witnessed antigen (rapid) test and symptom/COVID exposure check, then later receives risk estimate after all/most results collected

Model updated with individual data (age, vaccination status, location, antigen result etc)

Improved risk estimates communicated to ticket holders after individual data

Ticket holder passes symptom check, travels to event and and antigen test results collected, with

passes ID, CERTIFIC, contact tracing, ticket and security option to withdraw available

checks, receives safety reminders and attends event

Attendee travels home, limits contact and receives daily SMS reminders to isolate, report any symptoms and book post-event antigen test

Attendee takes antigen test, symptom check, attendee experience survey and receives completion reward

Model predictions compared with post event antigen test and questionnaire results

Evaluation of the validity of the risk model

1-2

weeks before event

2 days pre-event

Event day

Days 1-5 post- event

Days 6-8 post- event

Figure 1: CAPACITY process flow from the ticket holder perspective. On the left-hand side in red lined boxes are the timings of various stages in CAPACITY process flow. In the middle the process flow is described from the participant perspective. On the right-hand side, the interaction between user-supplied data and model-generated risk estimates is described. The certainty in the model output is conveyed through the varying shades of grey (the darker the colour, the more certain the model).

**Risk Communication.** The risk estimate is then provided to the participants using a variety of different formats, for better interpretability and communicability of the risk to the general public. Fig 2 shows a few examples of the displays used by the CAPACITY study.


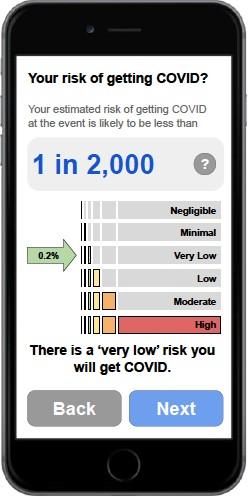

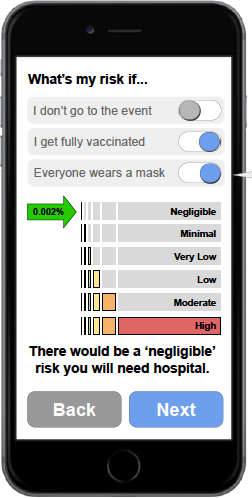

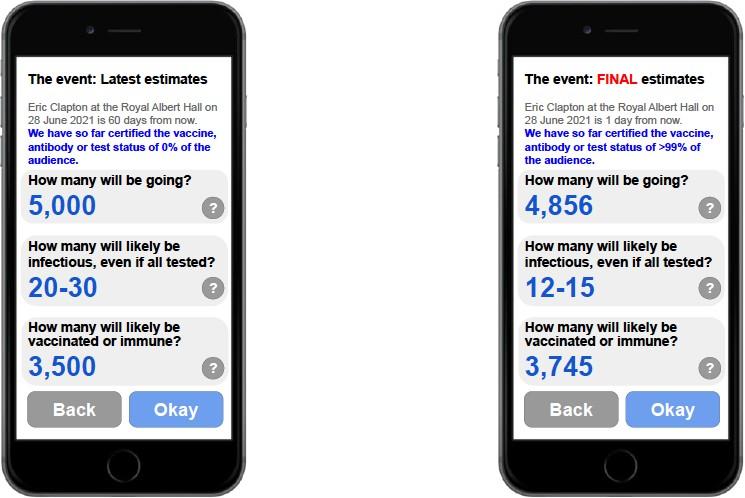


Figure 2: Top left – baseline estimates; Top right – final estimates; Bottom left – individual risk communication; Bottom right – tailored risk scores under different scenarios

**Dashboard.** The dashboard for event organizers is an R Shiny app that asks organizers to input relevant information on the event and generates predictions of the number of new infections that emerge from transmission during the event. In particular, the app predicts the numbers of infectious and susceptible people at the event using information provided on the location, date, and size of the event, and estimates relevant parameters in the aerosol transmission model using input data about the event space. Monte-Carlo simulations are then run by the app to estimate the number of new infections arising from transmission at the event, and simulation results are presented as a histogram to the user. Emphasis is placed on the median number of new infections, as well as worst-case scenarios (97.5^th^ and 99^th^ percentiles of new infections).


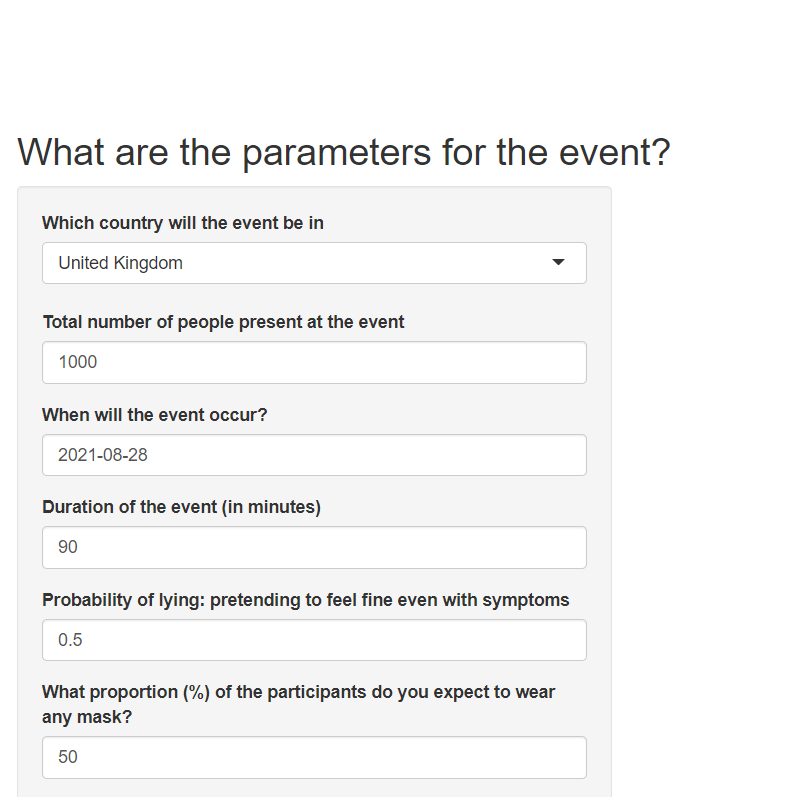


Figure 3: Input fields in the event planning dashboard


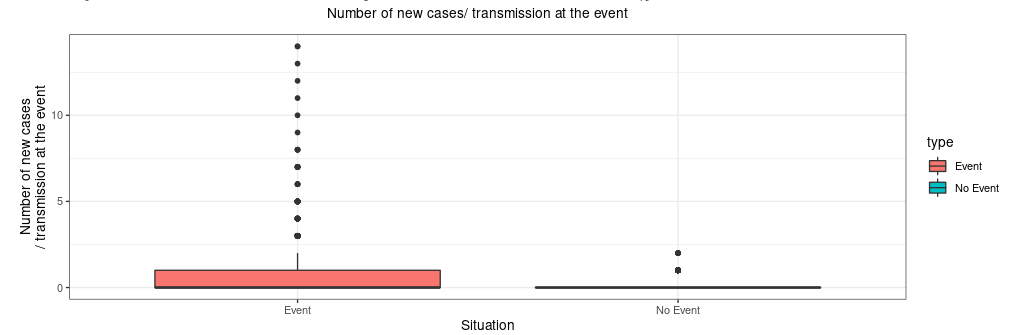


Figure 4: Sample simulation results for new infections during the event

**References:**

1. Parliament of the United Kingdom. Call for evidence in COVID-19 certification inquiry. 2021. <https://committees.parliament.uk/committee/327/public-administration-and-constitutional-affairs-committee/news/153049/call-for-evidence-in-covid19-certification-inquiry/>
2. Ada Lovelace Institute. What place should covid-19 vaccine passports have in society?, 2021. https://www.adalovelaceinstitute.org/summary/covid-19-vaccine-passports.
   Accessed: 11-08-2021.
3. Harris M, Kreindler J, El-Osta A, Esko, T, Majeed F. Safe management of full-capacity live/mass events in COVID-19 will require mathematical, epidemiological and economic modelling. Journal of the Royal Society of Medicine. 2021; 114(6):290-294. doi: 10.1177/01410768211007759.
4. Covid: Tests to be offered twice-weekly to all in England, BBC News. April 5, 2021.
   https://bbc.com/news/uk-56632084
