# Supplementary material for: Predicting COVID-19 Transmission to Inform the Management of Mass Events: Model-Based Approach
Source: JMIR Public Health Surveill. 2021 Dec 1;7(12):e30648. doi: 10.2196/30648 (PMC8638785; doi:10.2196/30648)
Supplement: Multimedia Appendix 5 [file publichealth_v7i12e30648_app5.docx]

# Model Validation

Model Validation is a crucial step in allowing this model to be deployed and trusted. In the previous appendices, we have run experiments on the prevalence estimation and vaccination status – allowing us to highlight and quantify the performance and reliability of these steps – but not on the transmission setting, due to the scarcity of available data. The transmission dynamics in our model are based on the Jimenez aerosol transmission model [1-3], which was retro-actively fitted from documented transmission events. This transmission step is in fact the most difficult one to validate: there are none or very few available datasets on COVID spread either following live events or tracking super spreader events, nor are there any statistics on how likely super spreader events are. As such, the majority of super spreader events that are documented currently (a) are generally not detailed enough to untangle the huge variability in context (outdoors vs indoors, activity performed, background prevalence, etc), and (b) suffer from selection bias --- and might not be reflective of the general distribution of events.

However, to overcome this hurdle and to try and make use of the currently available data, we propose the three following directions:

1. **Comparison with existing Super Spreader Event databases.** We begin our validation of the aerosol transmission model by using an openly available dataset with documented accounts of super spreader events [4]. This dataset is an open-source, manually curated database with now more than 1,600 Super-Spreading Events (SSEs) from around the world. These superspreading events are varied both in length (short events such as parties, extended co-living such as in dorms, prisons, or nursing homes), nature (sport participation, bar crawl, restaurant), or context (indoors, outdoor) to name a few. Despite the potential heterogeneity of this dataset and the selection bias that these events are likely to suffer from, we propose using this dataset as a way of validating the behaviour of our estimates, and their ability to model super spreader events. We begin by investigating the behaviour of the proportion of people infected at these events. To this end, we consider the events for which the number of participants and the total number of cases (before and after the event) are available. The following figure shows a plot of the percentage of people infected as a function of the event size. The blue line is the fitted loess line, along with its associated confidence interval. We note the strong spread of the percentage of people infected --- going anywhere from a few to 100% and yielding the following conclusion:
   *Lesson 1: In super spreader events (SSEs), the number of people infected is highly volatile. In particular, for small events, 100% secondary infections is not an unlikely scenario. The percentage of the crowd infected decreased however the size of the crowd.*


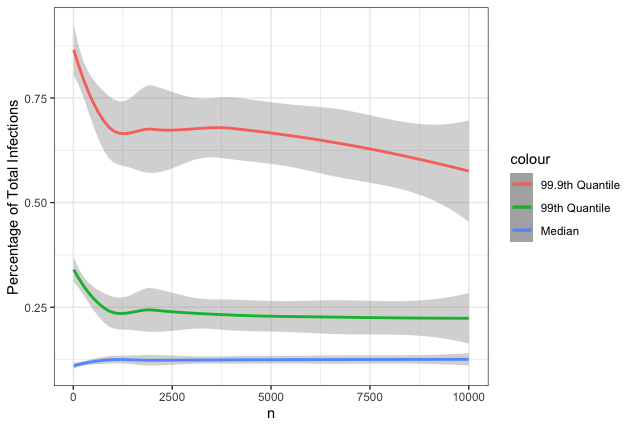

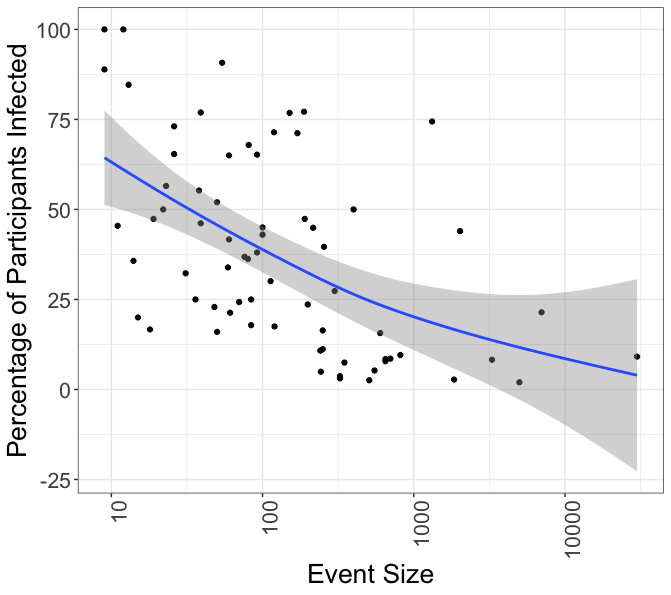


Figure E2 Simulation on the percentage of secondary cases according to our model .

Figure E1: Percentage of Participants infected as a function of the event size. The blue line is the fitted loess function, with associated confidence interval.

**Model sanity check nº1:**  We check if our current model behaves the same. Considering a scenario where 10% of the participants are initially infected, we vary the number of people at the event (whilst keeping the same density as our event in the RAH --- which we believe to be a reasonable approximation: larger events are not necessarily more dense than small gatherings), and check that we do observe a decreasing relationship between event size and proportion of secondary cases. The following figure highlights the result of 10,000 simulations per event size. It is reassuring to see that the upper quantiles (ie, the 99.9th quantiles) of these distributions (which correspond to potential super spreader events) follow the same decreasing behaviour as observed in the SSE dataset.

**Model sanity check nº2:** As a way of further assessing the validity of our model, we further filter the dataset for the recorded events for which the number of Index cases and the total number of secondary cases is available: this allows us to look at the transmissions at the event itself, this disentangling high transmissions due to high prevalence or high number of index cases. This results in a dataset of 26 indoor events. To limit the heterogeneity of the dataset, we stratify the events into two categories: “close” transmission settings (where the participants are expected to be in close proximity to one another, or a density less than 1 participant per square meter. This is meant to mimic scenarios such flights, or meal sharing), and “medium” (where the participants are farther apart --- such as in an office, or a density more than 1 participant per 9 square meter). These rates are displayed in Figure 3 (blue line denotes the fitted loess, along with confidence interval). We note again the high spread of the number of secondary cases--- indicating the high stochasticity of superspreading. We then proceed to assess whether our model would be able to account for these events. Using the ventilation parameters from the RAH example (which we think to be standard) and the two aforementioned densities, we simulate events of 90 minutes with the same number of participants and index cases. The purpose of this experiment is to check if the number of infections is achievable by the model. Note that this is again a sanity check, rather than a real statistical test. The super spreader events in this dataset are far too heterogeneous (in density, length. etc). Stratifying events according to close/medium proximity attempts to limit some of this heterogeneity.


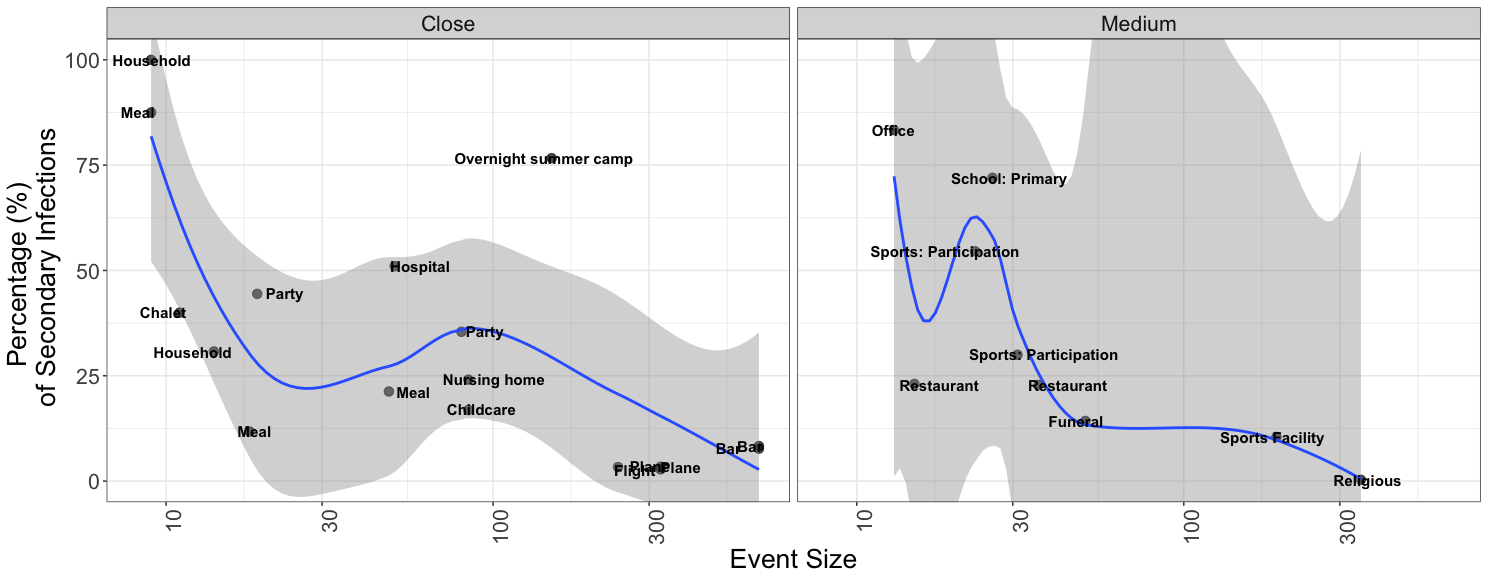


Figure 3: Proportion of Secondary Cases as a function of event size, stratified by proximity of the participants.

Using 10,000 simulations, we obtain the distributions highlighted in Figure 4. Most of these events are indeed qualified as super spreaders by our model--- which is reassuring. This does allow us to conclude that the numbers offered by our model are not unreasonable: most of the events in this dataset are indeed outliers according to our model, but they also fall within the realm of the model’s outputs.


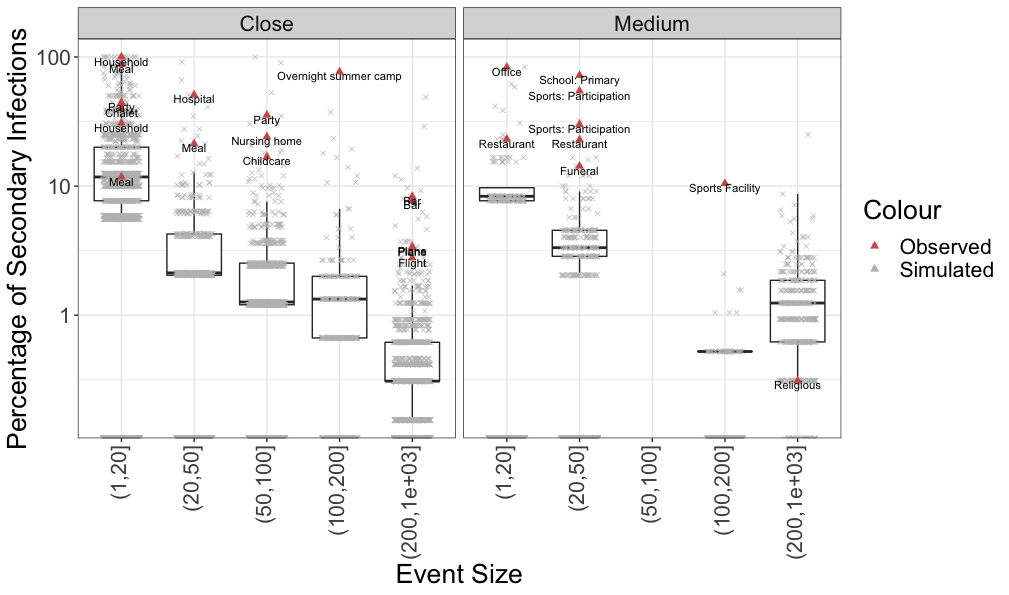


Figure 4: Observed vs simulated percentage of secondary infections occurring at potential super spreader events. The figure is faceted into two categories of events where the likely distance between people is ~1m (Close) or >1m (Medium). Events are further grouped on the x axis by the number of attendees. The percentage of people infected at the event (secondary infections) is indicated on the y axis with grey crosses indicating simulation results, and red triangles indicating observed cases.

1. **Benchmarking using the first live event studies:** In terms of randomised controlled trials or planned observational studies, we are aware of only of three that have been performed with sufficient information to validate or infirm our model:
   1. ***The Barcelona Winter concert:***  Revollo et al.[5] reported that in a randomised controlled trial for a live indoor music event held in Barcelona on December 12th, 2020,  0 out of 465 in the experiment (live-event) arm were Ag-RDT positive, and 2 out of 495 in the control (no-event arm).Their screening process is roughly similar to the one used by CERTIFIC.  We use this documented event to benchmark our model as follows:
      1. *Prediction scenario:* assuming that we wanted to predict all transmissions for such an event 4 weeks in advance. We run our prediction scenario for this indoor event, using the parameters described by the authors. According to these simulations, around 9.2 participants should have been infected, with an average of 0.56 infected participants passing through the screening process and entering the event. The number of predicted transmissions was 0.21 (CI: 0-2), which is in line with what was observed at the event.
      2. *Transmission validation:* We now replace the prevalence imputation part by the observed data, and focus on analyzing the transmission outcomes. On average, 5.89 should have been infected prior to the event, of which 4.98 should have been detected. The observed number of detections (0) among participants are lower than expected: the probability that no participant is detected as positive is 0.022 according to our model. This could be due to response/selection bias in the study cohort. This also indicates that our estimates (which assume the public to be sampled from the general Spanish public) will be conservative. Despite being conservative, the lack of transmission falls again within what is predicted by our pipeline – thereby validating our model outputs.
   2. ***The Barcelona Spring concert:*** Llibre et al. [6] observed 6 out of 5,000 Ag-RDT positives before a live music event held in Barcelona on March 27th, 2021 and 6 out of 4584 within 2 weeks after the event. Their screening process is roughly similar to the one used by CERTIFIC. We run our model on this benchmark. Using the observed incidence, we predict that an average of 8.47 should have been detected as positive by the screening process –  so that detecting exactly 6 individuals (as occurred in this event) has probability 0.1 and falls within the realm of likely observations. Moreover, this study shows that a total of 6 participants tested positive for COVID within 2 weeks of the event: 3 of which were likely to have been contaminated through other index cases, and one woman who was likely to have been in her incubation phase during the concert. The transmission source for the remaining two was not identified, and could stem from outside contamination or transmission at the concert. As such, supposing that the worst case total number of infectious participants at the event was likely to be 4 participants or more (probability of 0.04 in our model), the probability that it would result in 2 infections or less is  0.98 in our model. As such, this event falls within the realms of likely possibilities according to our model – thereby providing further reassurance as to the validity of the model.
   3. ***Prospective data: The CAPACITY Study, with one event to date: Standon Calling***. In a recent collaboration between Imperial College London, CERTIFIC and the Standon Calling family music festival (22-25 July 2021) an at-home, video-testing certification process was piloted resulting in clinically-certifiable LFT test results from CERTIFIC- a provider of services in line with standards overseen by a UK Government authorised private testing provider. 15,612 tickets were sold, and the festival had a total duration of four days. 40 participants tested positive upon entry, 10 of which were likely false positives. Data is currently being collected, and post-participation surveys sent out to assess the magnitude of transmission events. We plan on using this event as well as subsequent events ran by CERTIFIC as a way of further validating the model.

Note that these are only a few (two) realizations of an inherently volatile, stochastic process –– with high heterogeneity in conditions (outdoors, indoors, etc.). The power of this analysis can only be very limited: currently, this simply serves as another reassurance/“sanity check” that our model manages to produce estimates that are likely, rather than as an actual statistical validation step. However, as the number of live events increase, we hope to be able to add more data to corroborate the validation of our pipeline.

**c. Active Data Collection.** The lack of data is currently a real issue, that can only be overcome by starting a rigorous data collection process – one that is amenable to help calibrating the transmission process, and that must include information about the event (number of people, density, etc.).  To this end, we have added a questionnaire that people can fill in on our R-Shiny app [7], and that would help us expand the number of gathering/ live events. We are hoping that event organizers and individuals (involved in planning ceremonies, weddings, any type of gatherings) could look at our risk estimates, and volunteer to submit their data – thereby allowing a more scalable data collection process. In fact, one of our objectives in publishing our model and making it easy to access is to encourage the data collection that is so desperately needed for risk assessment, validating risk calculators and improving current transmission models.

**References:**

1. Jimenez. Aerosol transmission model, 2020. Available at <https://docs.google.com/spreadsheets/d/16K1OQkLD4BjgBdO8ePj6ytf-RpPMlJ6aXFg3PrIQBbQ/edit#gid=519189277>, Accessed August 3^rd^ 2021.
2. Miller SL, Nazaroff WW, Jimenez JL, et al. Transmission of SARS-CoV-2 by inhalation of respiratory aerosol in the Skagit Valley Chorale superspreading event. Indoor air. 2021; 31(2):314-323. doi: 10.1111/ina.12751.
3. Elbanna A, Wong GN, Weiner ZJ, et al. Entry screening and multi-layer mitigation of COVID-19 cases for a safe university reopening. medRxiv. Preprint posted September 2, 2020. doi: [10.1101/2020.08.29.20184473](https://doi.org/10.1101/2020.08.29.20184473).
4. Swinkels, K. (2020). SARS-CoV-2 Superspreading Events Around the World [Google Sheet]. Retrieved from [www.superspreadingdatabase.com](http://www.superspreadingdatabase.com). [Accessed 10-23-2021]
5. Revollo, Boris, et al. "Same-day SARS-CoV-2 antigen test screening in an indoor mass-gathering live music event: a randomised controlled trial." The Lancet Infectious Diseases (2021). doi: :https://doi.org/10.1016/S1473-3099(21)00268-1
6. Llibre, Josep M., et al. "Screening for SARS-CoV-2 antigen before a live indoor music concert: an observational study." Annals of Internal Medicine (2021). doi: 10.7326/M21-2278
7. COVID event risk survey Available at <https://docs.google.com/forms/d/e/1FAIpQLSfCqmEltbJtOhfVTd_yNvhu4t0yulyAziuxStGXx8YI0MVQ0w/viewform?usp=sf_link> [accessed August 16, 2021].
